# ELK1-mediated YTHDF1 drives prostate cancer progression by facilitating the translation of Polo-like kinase 1 in an m6A dependent manner

**DOI:** 10.7150/ijbs.75063

**Published:** 2022-10-18

**Authors:** Peizhang Li, Yuanping Shi, Dajun Gao, Huan Xu, Yun Zou, Zhong Wang, Wenzhi Li

**Affiliations:** 1 Department of Urology, Shanghai Ninth People's Hospital, Shanghai Jiao Tong University School of Medicine, Shanghai, China.; 2 Department of Endocrinology and Metabolism, Peking University People's Hospital, Beijing, China.; 3 Shanghai Key Lab of Tissue Engineering, Shanghai, China; 4 Department of Urology, Shanghai General Hospital, Shanghai Jiao Tong University School of Medicine, Shanghai, China.

**Keywords:** prostate cancer, N6-methyladenosine, YTHDF1, PLK1, ELK1

## Abstract

**Background**: N6-methyladenosine (m6A) is one of the most prevalent mRNA modifications in mammals, and it regulates the fate of modified RNA transcripts. In the current study, we aimed to elucidate the role of YTH m6A RNA-binding protein 1 (YTHDF1), a “reader” of m6A modification, in prostate cancer tumorigenesis.

**Methods**: We employed a multi-omics approach to detect the direct target of YTHDF1 upon manipulation of YTHDF1 expression in prostate cancer cells. Expression of YTHDF1 was also evaluated in human prostate tumors and either adjacent or paired normal tissues. Additionally, in vivo tumor growth and metastasis experimental assays were performed to evaluate the role of YTHDF1 in tumorigenesis. Finally, luciferase reporter assays and Chromatin immunoprecipitation (ChIP) were conducted to elucidate the transcriptional regulators of YTHDF1.

**Results**: We demonstrated that polo-like kinase 1 (PLK1) is a direct target of YTHDF1. YTHDF1 facilitated the translation efficiency of PLK1 in an m6A-dependent manner by identifying the m6A-modified PLK1 mRNA and subsequently promoted the hyperactivation of the PI3K/AKT signaling pathway. Moreover, our results indicated that YTHDF1 was upregulated in prostate cancer tissue and that high YTHDF1 expression was associated with adverse prognosis in patients with prostate cancer. Furthermore, upregulation of YTHDF1 promoted prostate cancer tumorigenesis and metastasis in vitro and in vivo. Additionally, dysregulation of ETS transcription factor ELK1 activated the transcription of YTHDF1 by directly binding to its promoter region.

**Conclusions**: Collectively, our findings suggest that the ELK1/YTHDF1/PLK1/PI3K/AKT axis is critical for prostate cancer progression and may serve as a potential therapeutic target for prostate cancer treatment.

## Introduction

Prostate cancer is one of the most common non-cutaneous cancers in men worldwide 1. It is also the second common cause of cancer death in United States (US) 2. Androgen deprivation therapy (ADT) is the first-line therapy for prostate cancer and has demonstrated improved overall survival (OS) in men diagnosed with metastatic prostate cancer 3, 4. However, the tumor may subsequently develop resistance to ADT and inevitably progress into castration-resistant prostate cancer (CRPC) 5. Metastasis is the primary cause of death in patients with prostate cancer. Although many potential therapeutic targets have been reported 6-8, it is crucial to identify new molecular targets that halt or slow disease progression.

N6-methyladenosine (m6A) is one of the most prevalent post-transcriptional mRNA modifications and has been reported to play an important role in tumorigenicity in various types of cancers 9-13. RNA modifications are installed by the m6A methyltransferase complex (“writer”), which is composed of METTL3 (a catalytic enzyme), METTL14 (an allosteric activator) 14, and the regulator, Wilms tumor 1 associated protein (WTAP) and are reversed by m6A demethylases (“eraser”), FTO and ALKBH5 15, 16. The m6A-modified RNA is recognized and influenced by m6A-specific binding proteins (“reader”) composed of YTH domain proteins, HNRNP family proteins, and IGF2BP family proteins to regulate pre-mRNA processing, including degradation and translation processes 17-21. To date, several studies have indicated that m6A modification is involved in many biological processes and diseases 22. Emerging evidence demonstrates that m6A modification is dysregulated in cancer cells and tumors and plays a critical role in controlling oncogene expression 9, 23. Therefore, the m6A machinery has become a promising therapeutic target for anticancer drug development. Exploring the regulatory mechanisms of RNA m6A modifications in prostate cancer progression is of great significance for improving the prognosis of patients with prostate cancer.

As a main cytoplasmic m6A reader, YTH domain family 1 (YTHDF1) has been reported to promote translation of target transcripts by recruiting translation initiation factors that affect tumor progression in several cancers 24. For instance, YTHDF1 was overexpressed in gastric cancer, and YTHDF1 deficiency impaired gastric cancer progression and metastasis. Mechanistically, YTHDF1 was reported to regulate the translation of FZD7 to activate Wnt/β-catenin signaling 25. Moreover, overexpression of YTHDF1 was associated with poor prognosis of ovarian cancer. YTHDF1 affected EIF3C translation in an m6A-dependent manner to promote ovarian cancer progression and metastasis 26. Compared with wild-type mice, Ythdf1-deficient mice showed antigen-specific CD8+ T cell antitumor response by inhibiting the translation of lysosomal cathepsins 27. However, the oncogenic role of YTHDF1 in prostate cancer remains unclear.

ETS transcription factor ELK1 is a number of the ETS family and ternary complex factor (TCF) subfamily. ELK1 is reported to play a significant role in the regulation of cell growth, differentiation, and survival 28. In addition, ELK1 directly bind to AR to upregulate a major subset of its target genes which is essential to prostate cancer cell growth and survival 29. However, novel regulatory mechanism of ELK1 in prostate cancer was not fully elucidated.

Here, we report significant upregulation of YTHDF1 in prostate cancer tissues. Knocking out YTHDF1 inhibited proliferation, migration, and invasion of prostate cancer cells *in vitro* and *in vivo*. Mechanistically, we identified the target genes of YTHDF1 which are involved in prostate cancer progression. Moreover, YTHDF1 regulated prostate cancer growth and metastasis via increasing translational efficiency of polo-like kinase 1 (PLK1) in an m6A dependent manner, and the transcription of YTHDF1 was activated by the dysregulation of ELK1 in prostate cancer. Thus, our results demonstrated the prominent oncogenic role of the m6A reader YTHDF1 in prostate cancer development.

## Materials and method

### Tumor samples

The study design and experiments were approved by the approval of the Ethics Committee of the Shanghai Ninth People's Hospital. Fresh frozen samples of prostate tumor and adjacent normal tissues were collected and examined; The histology of prostate tumor was confirmed by experienced pathologists. Study participants provided written informed consent. The clinical information of the patients with prostate cancer is shown in Table 1. Scoring of immunohistochemistry (IHC) staining was based upon the staining intensity (I score: negative, 0; weak, 1; moderate, 2; and intense, 3) and the percentage of positive stained cells (P score: 0-5%, score of 0; 5-25%, score of 1; 25-50%, score of 2; and 50-75%, score of 3; >75%, score of 4) to obtain a final score (I score × P score). Two senior pathologists performed the scorings independently in a blinded manner.

### Cell culture

Human prostate cancer cell line, PC-3, originated from bone marrow metastases in a 62-year-old white male patient diagnosed with grade IV prostate cancer. Prostate cancer cells DU145 were established from the brain metastasis of a 69-year-old Caucasian patient with prostate cancer. The cells were obtained from the National Collection of Authenticated Cell Cultures at the Chinese Academy of Science (Shanghai, China). PC-3 and DU145 cells were cultured in Minimum Essential Media (MEM; Gibco) supplemented with 10% fetal bovine serum (FBS; Gibco). Cell authentication was validated by short tandem repeat (STR) profiling.

### Establishment of stable knockout cell lines

Lentiviral vector LV120-pHBLV-U6-gRNA-EF1-fluc-T2A-Blasticidin, obtained from Hanheng Biotechnology (Hanheng Biotechnology Co., Ltd., Shanghai, China), was used to express gRNAs in PC-3 and DU145 cell lines. For lentivirus production, HEK293T cells were co-transfected with lentiviral vector and the packaging vectors psPAX2 and pMD2.G using Lipofectamine 2000 (Invitrogen) according to the manufacturer's instructions. Supernatant was collected at 48 h post transfection and, after implementing it with 10 µg/mL polybrene, added to PC-3 and DU145 cells. After 48 h incubation, the medium was replaced with fresh medium containing 10 μg/mL Blasticidin for selection of infected cells and generation of the stable KO cell lines. The gRNAs and shRNAs sequences are listed in Supplementary Table 5.

### RNA isolation and RT-qPCR

Total RNA was extracted from prostate cancer cells using TRIzol reagent (Invitrogen), according to the manufacturer's protocol. Total RNA was then reverse transcribed to cDNA using random primers and a Revert Aid First Strand cDNA Synthesis kit (Thermo Fisher, USA). qRT-PCR was conducted using TB Green Premix Ex Taq (Takara) on a Bio-Rad CFX96TM Real-Time PCR System (Bio-Rad). GAPDH was used as an internal control. RT-qPCR primers were synthesized by BioSune (Shanghai, China) and are listed in Supplementary Table 1.

### Western blotting

Total proteins were extracted using RIPA lysis buffer (Sangon Biotech, China) containing 1% protease inhibitor cocktail (CST, USA). After separation on a 10% polyacrylamide gel, and separation via sodium dodecyl sulfate-polyacrylamide gel electrophoresis (SDS-PAGE), the protein extracts were transferred onto a polyvinylidene fluoride (PVDF) membrane and placed in 5% BSA for blocking at room temperature for 1 h. Subsequently, the membranes were incubated with the primary antibody. Finally, the membranes were incubated with secondary antibodies, and the proteins were visualized using ECL Prime. Antibody details are listed in Supplementary Table 2.

### Cell proliferation assays

Cell proliferation was measured using the CCK-8 assay (KeyGEN, Nanjing, China) following the manufacturer's instructions. YTHDF1-KO or YTHDF1-overexpression prostate cancer cells were seeded into 96-well plates at a density of 3,000 cells per well, and cultured overnight. The cells were treated with CCK-8 solution for 1 h. The number of viable cells was measured at 450 nm using a microplate reader every 24 h for 3 days.

For the colony formation assay, treated PC-3 and DU145 cells were seeded into 6-well plates (at a density of 200 cells per well). The cells were cultured in a humidified atmosphere containing 5% CO2 at 37 °C for two weeks. After washing with PBS and fixing with 4% PFA, 0.1% crystal violet staining was performed to observe the colonies. Visible colonies were then manually counted.

### Wound healing assays

The migration ability of prostate cancer cells was measured using wound-healing assays. First, the cells were cultured to confluency. A wound was created by scraping the cell monolayer with a 10 µL plastic pipette tip, and then the cells were cultured in medium without FBS. Images were captured immediately after the scratch and again 24 or 36 h later. Cell migration was quantitatively estimated by measuring the gap width.

### Transwell migration and invasion assays

For transwell invasion and migration assays, PC-3 and DU145 cells infected with overexpression virus or knockout virus were seeded into 6-well plates. After transfection for 48 h, the cells were collected and suspended in serum-free medium, and 2 × 10^4^ cells were plated into 24-well plates (Corning Costar Corp) according to the manufacturer's instructions. For the migration assays, the cells were placed in the upper chambers without coated membranes. For invasion assays, membranes of the upper chambers were coated with 1:5 diluted Matrigel (BD Biosciences, USA). All lower chambers were incubated with 500 µL medium containing 20% FBS. After incubation for 18 h for the migration assays and 24 h for the invasion assays, the cells on the lower surface were fixed and stained with 0.1% crystal violet solution. Subsequently, migrated or invaded cells were imaged using a bright light microscope, and cell number was counted using the ImageJ software.

### *In vivo* experiments

Nude mice (4‒6 weeks old) were purchased from Shanghai Jie Si Jie Laboratory Animal Ltd. and maintained at the animal care facility of the Experimental Animal Center of National Dong-Hua University in SPF barrier facilities. YTHDF1 stably overexpressing or YTHDF1-KO luciferase-labeled PC-3 cells were re-suspended in 0.1 mL PBS and injected subcutaneously into the flanks of BALB/c nude mice. The volume of the tumor was measured every 5 days according to the formula: volume = 0.5 × length × (width)^2^. At the end of the experiment, the tumors were surgically dissected and collected for further study. For the metastasis assays, 1 × 10^6^ cells were inoculated into the tail vein of male BALB/c nude mice. The IVIS Spectrum animal imaging system was used to examine tumor metastasis.

### RNA sequencing (RNA-seq)

Total RNA was isolated and purified from YTHDF1-knockout or control PC-3 cells using TRIzol reagent (Invitrogen) according to the manufacturer's procedure. Poly (A) RNA was purified from 1μg total RNA using Dynabeads Oligo (dT)25-61005 (Thermo Fisher, CA, USA) using two rounds of purification. Then, we performed the 2×150bp paired-end sequencing (PE150) on an illumina Novaseq™ 6000 (LC-Bio Technology CO., Ltd., Hangzhou, China) following the vendor's recommended protocol. HISAT2 (https://ccb.jhu.edu/software/hisat2) was utilized to map reads to the reference genome of Homo sapiens GRCh38. The differentially expressed mRNAs were selected with fold change > 2 or fold change < 0.5 and with P value < 0.05 by R packages EdgeR (https://bioconductor.org/packages/release/bioc/html/edg-eR.html).

### m6A sequencing (m6A-seq)

Total RNA was isolated and purified using TRIzol reagent (Invitrogen, USA) and the RNA amount of each sample was quantified using NanoDrop ND-1000 (NanoDrop, USA). Then, Poly (A) RNA is purified from 30μg total RNA using Dynabeads Oligo (dT)25-61005 (Thermo Fisher, USA). After incubated for 2h at 4℃ with m6A-specific antibody (Synaptic Systems, cat.202003, Germany) in IP buffer (50 mM Tris-HCl, 750 mM NaCl and 0.5% Igepal CA-630), the IP RNA was reverse-transcribed to create the cDNA by SuperScript™ II Reverse Transcriptase (Invitrogen, cat.1896649, USA). The average insert size for the final cDNA library was 300±50 bp. At last, we performed the 2×150bp paired-end sequencing (PE150) on an illumina Novaseq™ 6000 (LC-Bio Technology Co., Ltd., Hangzhou, China).

### RNA immunoprecipitation and high-throughput sequencing (RIP-seq)

Cells seeded in a 15cm dish at 80% confluency were cross-linked by ultraviolet light at 254nm (200J/cm2), then harvested and lysated. RNA immunoprecipitation (RIP) assay was performed with a Magna RIP RNA Binding Protein Immunoprecipitation Kit (Millipore, USA), using antibody specific for YTHDF1 (ab220162, Abcam). Input and co-immunoprecipitated RNAs were extracted with according to the instructions of the RNeasy Mini kit (QIAGEN, Germany). RNA-seq libraries were generated with the NEBNext Ultra™ RNA library Prep Kit (NEB E7770S) and were subjected to quality validation using the Agilent Bioanalyzer 2100. Then, the cDNA libraries were sequenced using Illumina platforms via a 2x150bp paired-end sequencing protocol.

### TMT labelling proteomic analysis

The peptide mixture was labeled with TMT10-plex reagents for 2 h at 25℃. The labeled peptide samples were then pooled and lyophilized in a vacuum concentrator. The peptides were re-dissolved in solvent A (A: 0.1% formic acid in water) and analyzed by online nanospray LC-MS/MS on Orbitrap Fusion™ Lumos™ Tribrid™ coupled to EASY-nLC 1200 system (Thermo Fisher Scientific, MA, USA). The column flow rate was maintained at 600 nL/min with the column temperature of 40°C. The electrospray voltage of 2 kV versus the inlet of the mass spectrometer was used. The mass spectrometer was run under data dependent acquisition mode, and automatically switched between MS and MS/MS mode.

### Polysome profiling

Prostate cancer cells were treated with cycloheximide (CHX) at 100 μg/ml for 2 minutes before collection. Cells were lysed on ice, and then centrifuged. Next, collect the supernatant and load onto a 10/50% w/v sucrose gradient. The gradients were centrifuged at 4°C for 4 hours at 27,500 rpm (Beckman, rotor SW28). Subsequently, the samples were fractioned and analyzed by Gradient Station (BioCamp) equipped with an ECONO UV monitor (BioRad) together with a fraction collector (FC203B, Gilson). The fractions were isolated total RNA by TRIzol reagent for RT-qPCR analysis.

### Chromatin immunoprecipitation (ChIP) assay

ChIP was performed as previously described 30. A total of 1 × 10^7^ cells were crosslinked in 1% formaldehyde for 10 min at 37 °C. Subsequently, the DNA was sheared to obtain 200-1000 bp fragments using sonication. Sonicated chromatin was diluted to a final concentration of 0.1% SDS. Then, the aliquots were incubated with anti-ELK1 antibodies or isotype control IgG for 2 hours. The immunoprecipitated DNA was retrieved from ChIP-Grade Protein G Agarose Beads (CST, 9007).

### Statistics analysis

All statistical analysis were performed using SPSS (22.0). The data are expressed as the MEAN±SD. Statistical analysis was performed by Student's t-test. Results were considered statistically significant with a p value < 0.05. Overall Survival (OS) and Progression Free Interval (PFI) was calculated using the Kaplan-Meier method, and the log-rank test was performed for comparisons of Kaplan-Meier curves.

## Results

### YTHDF1 is overexpressed in prostate cancer

**Figure 1:**
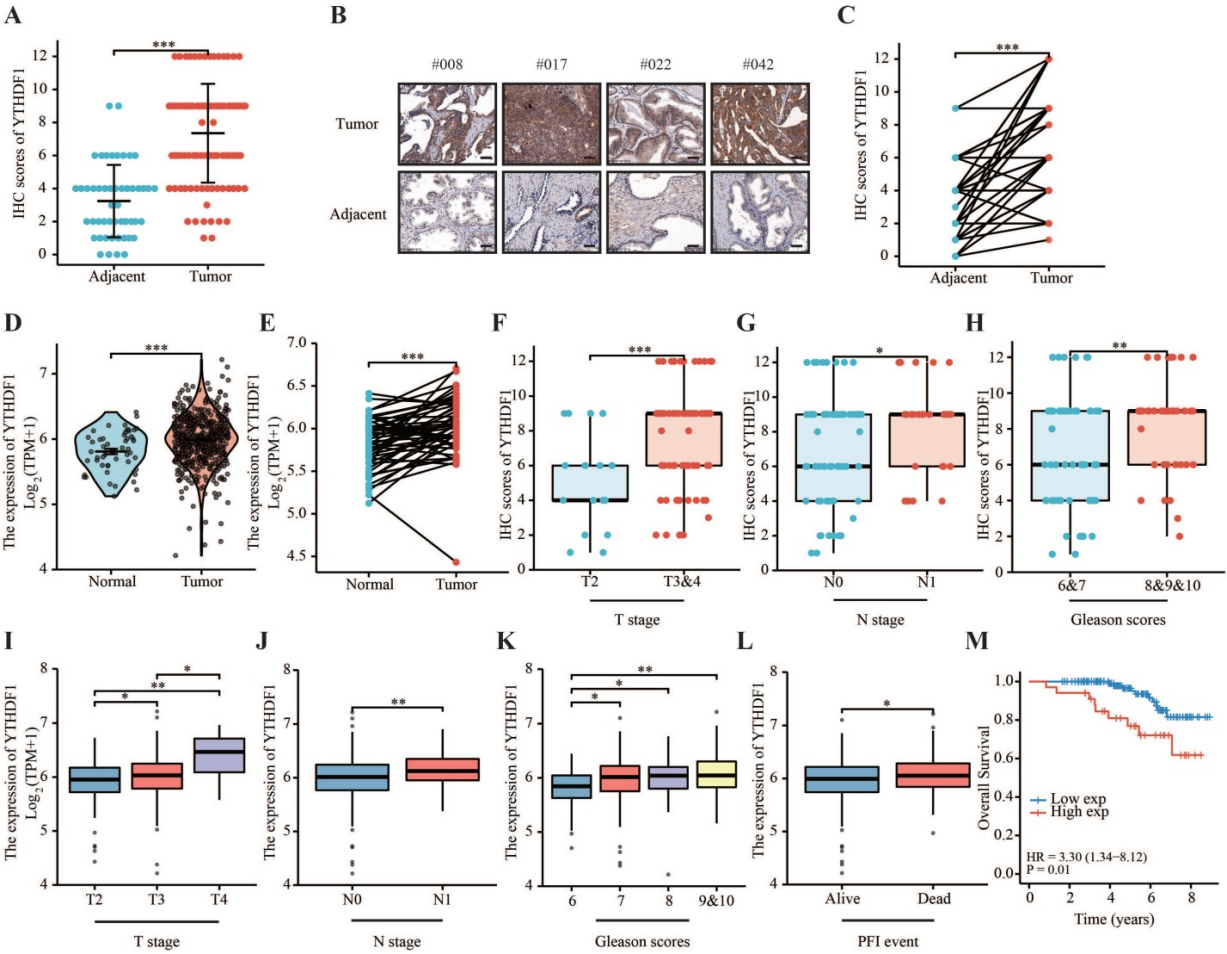
** YTHDF1 was highly expressed in prostate cancer and was related to a poor prognosis. (A)** Protein levels of YTHDF1 in prostate cancer and adjacent normal prostate tissues. **(B)** Representative immunohistochemical images of YTHDF1 expression in prostate cancer and normal prostate tissues (Scale bar: 50 μm).** (C)** Protein levels of YTHDF1 in prostate cancer and paired adjacent normal tissues. **(D)** RNA levels of YTHDF1 in prostate cancer and adjacent normal prostate tissues in The Cancer Genome Atlas (TCGA) database. **(E)** RNA levels of YTHDF1 in prostate cancer and paired adjacent normal prostate tissues in TCGA database. **(F)** Correlation between T stage and YTHDF1 expression. **(G)** Correlation between N stage and YTHDF1 expression. **(H)** Correlation between the Gleason score and YTHDF1 expression. **I** Correlation between T stage and YTHDF1 expression in TCGA database. **(J)** Correlation between N stage and YTHDF1 expression in TCGA database. **(K)** Correlation between Gleason score and YTHDF1 expression in TCGA database. **(L)** Correlation between progression free interval events and YTHDF1 expression in TCGA database. **(M)** Kaplan-Meier analysis of prostate cancer patients for correlation between YTHDF1 expression and overall survival. Data are indicated as mean ± standard deviation, ns P ≥ 0.05, * P < 0.05, ** P < 0.01, *** P < 0.001.

To elucidate the expression pattern of YTHDF1 in prostate cancer, two tissue microarrays (TMA) including 259 prostate tumor tissues and 50 tumor-adjacent normal tissues were immunostained for YTHDF1. Each tissue was assigned an immunohistochemistry (IHC) score on a categorical scale of 0 to 12. The results showed that YTHDF1 expression was upregulated in cancer tissue compared with normal prostate tissue, and a significant increase was also observed in prostate cancer compared to paired normal prostate tissue (Figure 1A‒C, Supplementary Figure S1A). Consistently, prostate cancer tissues had significantly higher YTHDF1 mRNA expression than normal tissues according to The Cancer Genome Atlas (TCGA) database (Figure 1D, E). In addition, RT-qPCR and western blotting analyses also confirmed the higher expression of YTHDF1 in prostate cancer cell lines (PC-3 and DU145) than in the normal prostate epithelial cell line (RWPE-1) (Supplementary Figure S2A, B). These data suggest that YTHDF1 expression is specifically elevated in prostate cancer. Next, we analyzed the correlation between YTHDF1 expression and clinicopathological characteristics of prostate tumors. YTHDF1 expression and other clinical features of the patients with prostate cancer are displayed in Table 1. The data indicated that a higher expression of YTHDF1 correlated with a higher pathologic T and N stages (Figure 1F, G). YTHDF1 expression tended to increase with increasing Gleason scores (Figure 1H). YTHDF1 expression and clinical features of prostate cancer patients in TCGA database are shown in Supplementary Table 3. Analysis of TCGA-PRAD database yielded similar results (Figure 1I‒L). Furthermore, Kaplan-Meier survival analysis showed that high YTHDF1 expression correlated with poor OS (Figure 1M, Supplementary Figure 1B). Univariate and Multivariate Cox regression analyses suggested that high YTHDF1 expression was independently related to poor overall survival (Table 2). These data suggest that YTHDF1 may be a diagnostic and prognostic biomarker for prostate cancer.

### YTHDF1 KO impaired tumorigenesis and metastasis of prostate cancer

**Figure 2:**
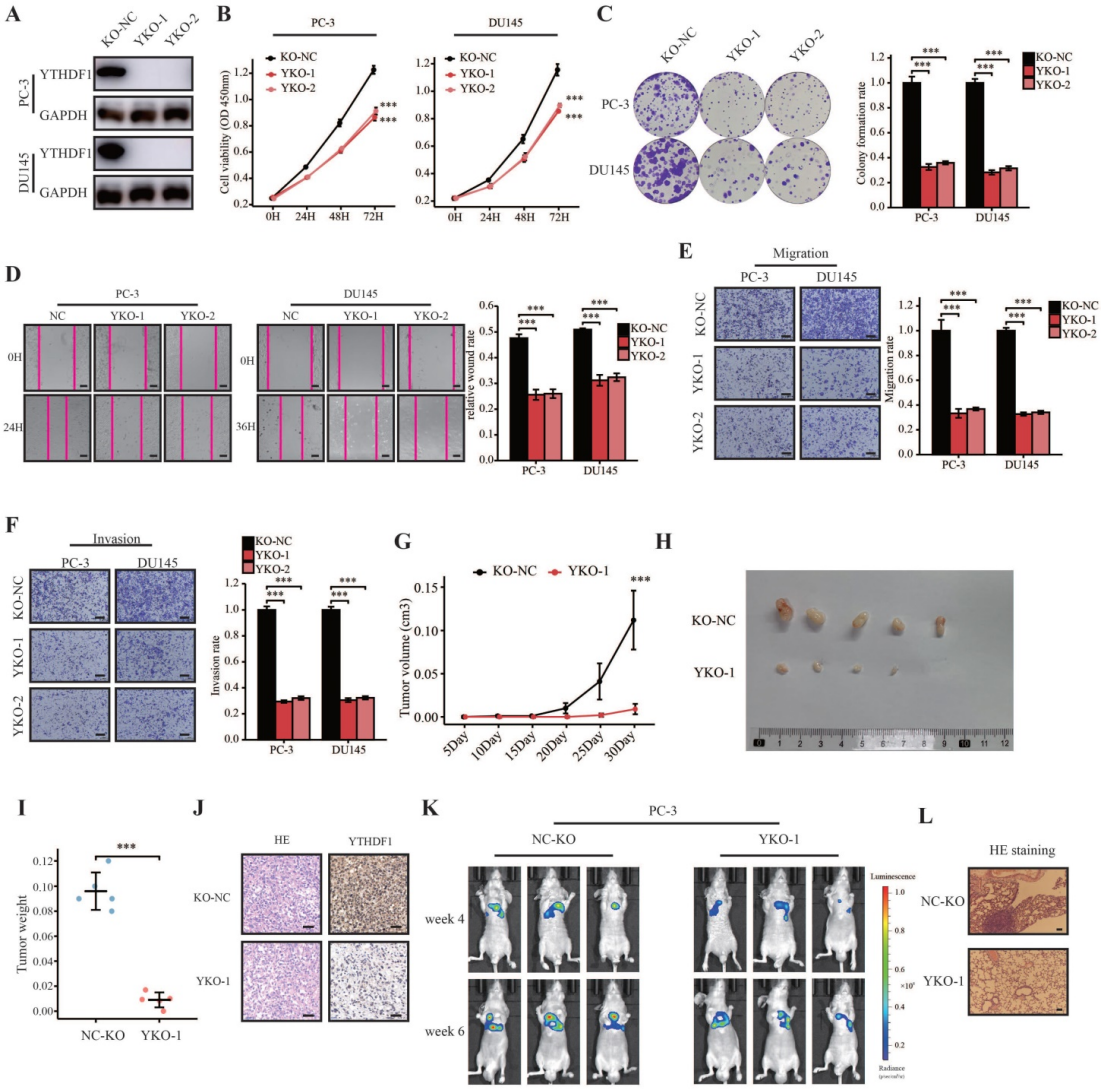
** YTHDF1-deficient impaired prostate cancer tumorigenesis and metastasis *in vitro* and *in vivo.* (A)** Western blot analysis showing the knockout efficiency of YTHDF1 in prostate cancer cells. **(B)** Cell viability analysis of YTHDF1-knockout prostate cancer cells using CCK-8 assays. **(C)** Analysis of colony formation ability in YTHDF1-knockout prostate cancer cells using colony formation assays. **(D)** Wound healing assay was performed to determine the migration of YTHDF1-deficient PC-3 and DU145 cells (Scale bar: 50 μm). **(E-F)** Transwell migration and invasion assays were conducted to determine the migration and invasion capacity of stable YTHDF1-knockout prostate cancer cells (Scale bar: 50 μm). **(G)** The tumor growth curve of xenografts was plotted in the negative control (KO-NC) and YTHDF1-knockout (YTHDF1-KO) groups (n = 5 in each group) by measuring the tumor size (0.5 × length × width^2^). **(H)** Images of xenograft tumors in each group (n = 5).** (I)** Weight of xenograft tumors in each group (n = 5). **(J)** Representative hematoxylin and eosin staining and immunohistochemistry forYTHDF1 in xenograft tumors of each group (Scale bar: 50 μm). **(K)** Bioluminescent imaging of BALB/c nude mice tail vein injection metastasis model with KO-NC and YTHDF1-KO luciferase-labeled PC-3 cells at weeks 4 and 6. **(L)** Representative hematoxylin and eosin staining photographs of metastatic prostate cancer in the lungs (Scale bar: 100 μm). Data are indicated as mean ± standard deviation, ns P ≥ 0.05, * P < 0.05, ** P < 0.01, *** P < 0.001.

To investigate the oncogenic role of YTHDF1 in prostate cancer, we established stable YTHDF1-knockdown PC-3 and DU145 cell lines. YTHDF1 knockdown was confirmed at both the mRNA and protein levels (Supplementary Figure S3A, B). YTHDF1 knockdown decreased cell proliferation and colony forming ability in prostate cancer cells (Supplementary Figure S3C‒E). Moreover, knockdown of YTHDF1 in both PC-3 and DU145 cells significantly suppressed their migration and invasion abilities (Supplementary Figure S3F‒H). To consolidate our findings, the CRISPR/Cas9 gene editing system was used to knock out YTHDF1 in PC-3 and DU145 cells, and the knockout efficiency was examined at the protein level using western blotting (Figure 2A). Consistent with the knockdown of YTHDF1 in PC-3 and DU145 cells, knockout of YTHDF1 significantly impaired cell proliferation and colony-forming ability in prostate cancer cells (Figure 2B, C). Furthermore, the YTHDF1-KO significantly inhibited prostate cancer cell migration and invasion (Figure 2D‒F).

To further evaluate the role of YTHDF1 in prostate cancer *in vivo*, we performed in vivo orthotopic implantation experiments where the cells were injected subcutaneously into the flanks of BALB/c nude mice. The results showed that YTHDF1-KO tumors grew slower than the negative control tumors (Figure 2G). Additionally, the tumor volume and weight in the YTHDF1-KO group were smaller than those in the control group (Figure 2H, I). IHC results confirmed that YTHDF1 was deficient in YTHDF1-KO tumors (Figure 2J). Next, we evaluated the impact of YTHDF1-KO on prostate cancer metastatic colonization using tail vein injection assay. The results indicated that the YTHDF1-KO significantly inhibited distant metastasis in mice injected with PC-3 cells (Figure 2K, L). In summary, YTHDF1-KO impaired the tumorigenesis and metastasis of prostate cancer.

### YTHDF1 promoted tumorigenesis and metastasis of prostate cancer

**Figure 3:**
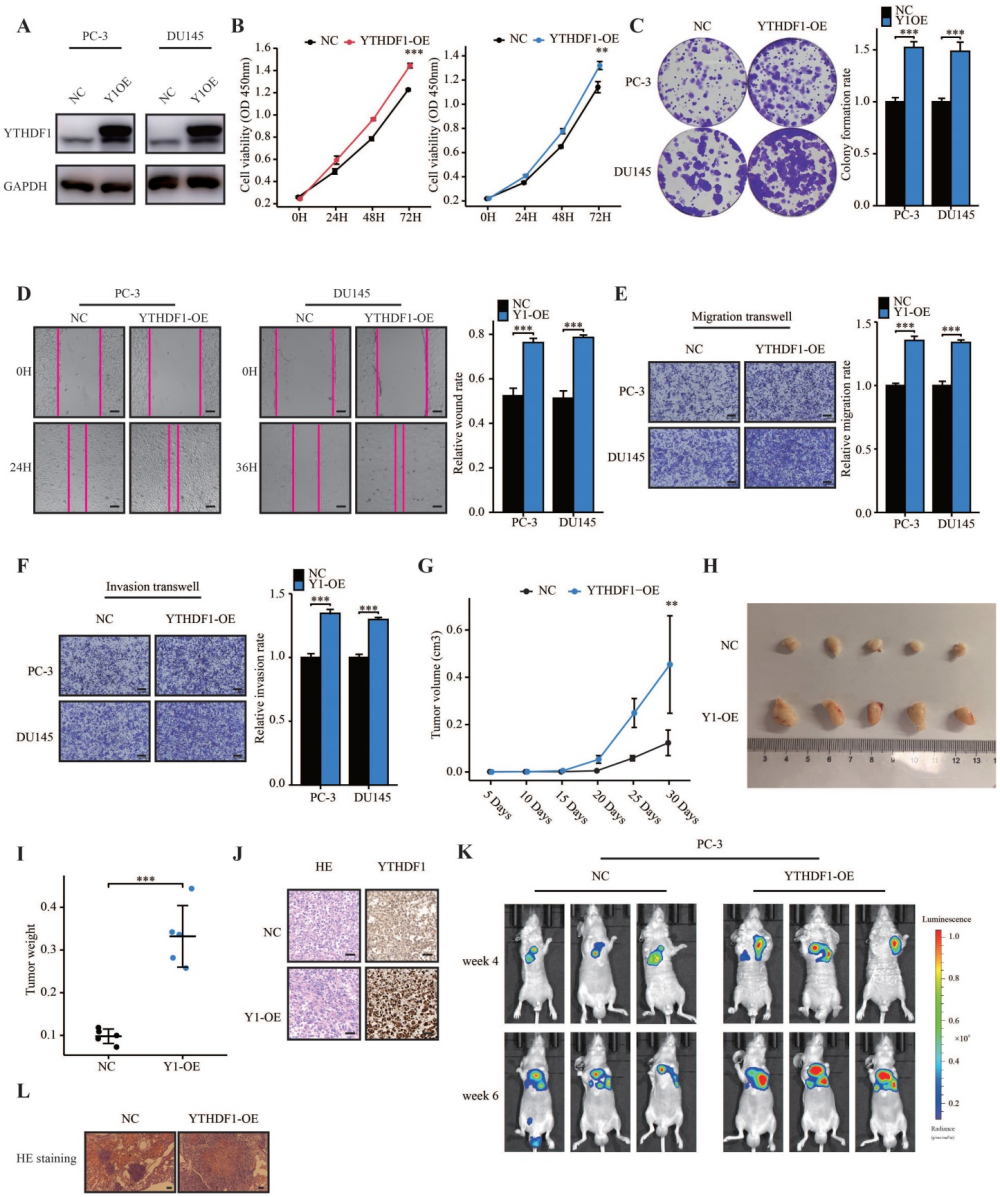
** YTHDF1 overexpression facilitated prostate cancer tumorigenesis and metastasis *in vitro* and *in vivo.* (A)** Western blot analysis showing the overexpression efficiency of YTHDF1 in prostate cancer cells. **(B)** Analysis of cell viability in YTHDF1-overexpression prostate cancer cells using CCK-8 assays. **(C)** Analysis of colony formation ability in YTHDF1-overexpression prostate cancer cells using colony formation assays. **(D)** Wound-healing assay was performed to determine the migration of YTHDF1-overexpression PC-3 and DU145 cells (Scale bar: 50 μm). **(E‒F)** Transwell migration and invasion assays were conducted to determine the migration and invasion capacity of stable YTHDF1 overexpressing prostate cancer cells (Scale bar: 50 μm). **(G)** The tumor growth curve of xenografts was plotted in negative control (NC) and YTHDF1-overexpressing (YTHDF1-OE) groups (n = 5 in each group) by measuring the tumor size (0.5 × length × width^2^). **(H)** Images of xenograft tumors in each group (n = 5). **(I)** Weight of xenograft tumors in each group (n = 5). **(J)** Representative hematoxylin and eosin staining and immunostaining for YTHDF1 in xenograft tumors of each group (Scale bar: 50 μm). **(K)** Bioluminescent imaging of BALB/c nude mice tail vein injection metastasis model with NC and YTHDF1-OE luciferase-labelled PC-3 cells at weeks 4 and 6. **(L)** Representative hematoxylin and eosin staining photographs of metastatic prostate cancer in the lungs (Scale bar: 100 μm). Data are indicated as mean ± standard deviation, ns P ≥ 0.05, * P < 0.05, ** P < 0.01, *** P < 0.001.

To further elucidate the role of YTHDF1 in prostate cancer, we established stable YTHDF1-overexpression PC-3 and DU145 cells and confirmed the YTHDF1 overexpression at the protein level (Figure 3A). YTHDF1-overexpression promoted cell proliferation and colony-forming ability of prostate cancer cells (Figure 3B, C). Moreover, upregulated YTHDF1 induced migration and invasion of prostate cancer cells (Figure 3D‒F). Additionally, the results of subcutaneous transplantation experiments showed that YTHDF1-overexpression tumors grew faster than negative control tumors (Figure 3G) and that tumor volume and weight in the YTHDF1-overexpression group were larger than those in the control group (Figure 3H, I). The protein expression of YTHDF1 was upregulated in YTHDF1-overexpression tumors according to IHC results (Figure 3J). To test directly whether YTHDF1-overexpression could have an effect on the metastatic capacity of prostate cancer, we performed an experimental model of metastasis where lung colonization of prostate cancer cells following tail vein injection was assessed. YTHDF1-overexpression PC-3 cells showed a significant increase in lung colonization capacity compared to the negative control cells (Figure 3K, L). In summary, these data demonstrated that YTHDF1-overexpression promoted the tumorigenesis and metastasis of prostate cancer.

### Identification of the YTHDF1 targets in prostate cancer

**Figure 4:**
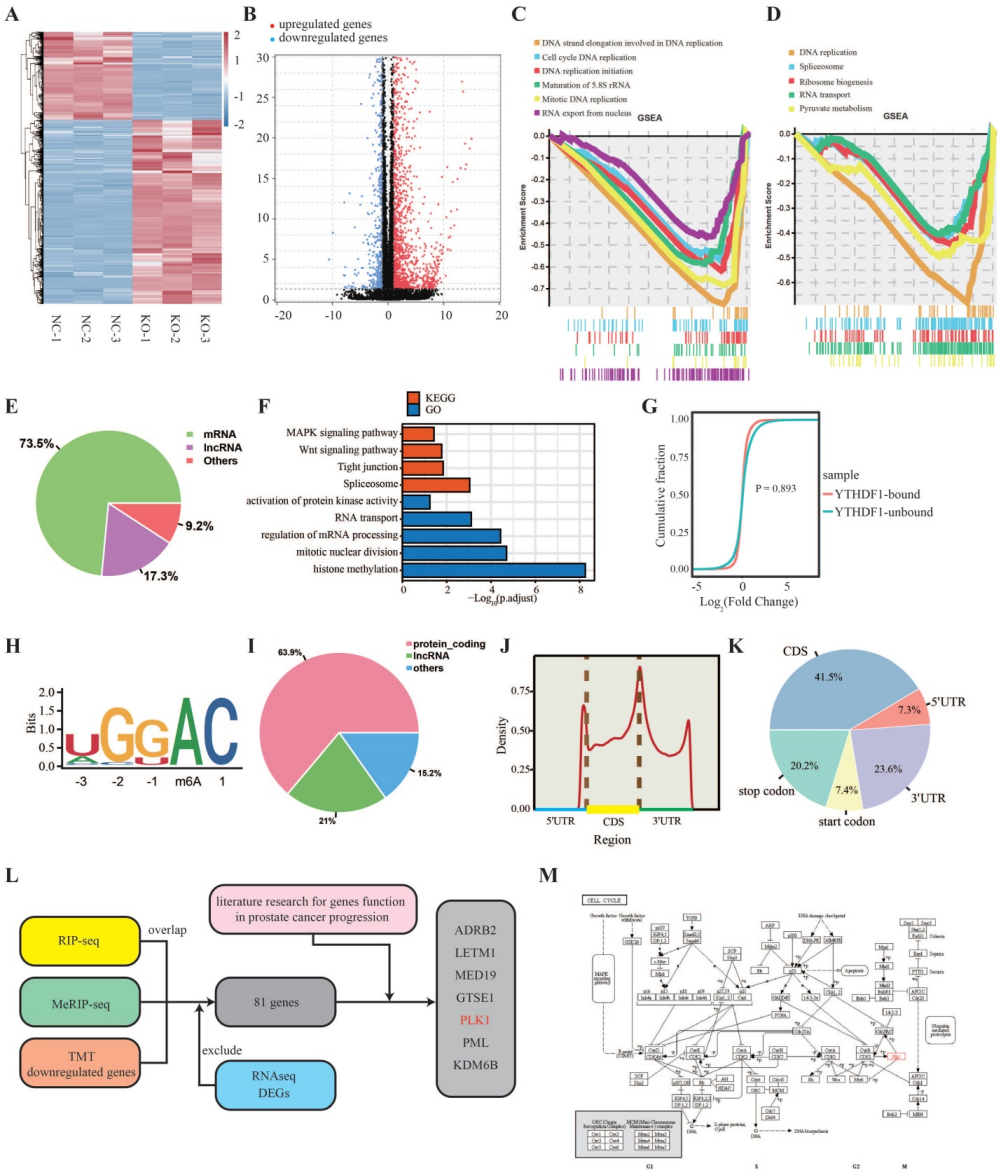
** Transcriptome-wide identification of the YTHDF1 targets in prostate cancer cells. (A)** Heatmap illustrating the differentially expressed genes (DEGs) in PC-3 cells between KO-NC and YTHDF1-KO groups. **(B)** Volcano map showing DEGs in PC-3 cells between the KO-NC and YTHDF1-KO groups. **(C)** Gene Set Enrichment Analysis (GSEA) plots showing the Gene Ontology of DEGs altered by knockout of YTHDF1 in prostate cancer cells. **(D)** GSEA plots showing the Kyoto Encyclopedia of Genes and Genomes (KEGG) pathway of DEGs altered by knockout of YTHDF1 in prostate cancer cells. **(E)** Distribution of immunoprecipitation RNAs in different RNA subgroups. **(F)** GO and KEGG enrichment analyses of immunoprecipitated genes. **(G)** Cumulative distribution map of YTHDF1-targeted and non YTHDF1-targeted genes expression.** (H)** Top consensus motif detected using HOMER with m6A-seq peaks in PC-3 cells. **(I)** Distribution of m6A modified RNAs in different RNA subgroups.** (J‒K)** Distribution of m6A modification locations in transcripts of PC-3 cells. **(L)** Schematic workflow of YTHDF1 downstream target analysis using m6A-seq, RIP-seq, TMT proteomic analysis, and RNA-seq. **(M)** KEGG annotated that PLK1 is involved in the G2-M phase of the cell cycle.

To elucidate the underlying mechanisms of YTHDF1 in prostate cancer progression, we performed RNA profiling of YTHDF1-KO and negative control PC-3 cells. Volcano plot and heatmap results revealed that 1,650 genes were upregulated and 790 genes were downregulated in YTHDF1-KO PC-3 cells at a fold change > 2 and p < 0.05 (Figure 4A, B, Supplementary Table 4). Gene set enrichment analysis (GSEA) indicated that the differentially expressed genes were enriched for DNA strand elongation in DNA replication and were involved in DNA replication-related pathways such as cell cycle, DNA replication initiation, maturation of 5.8S rRNA, mitotic replication, spliceosome, and ribosome biogenesis in eukaryotes (Figure 4C, D). These are key processes in prostate cancer tumorigenesis. RIP-seq was performed to screen for YTHDF1-binding genes in PC-3 cells. A total of 4,421 genes were immunoprecipitated, 3,249 (73.5%) of which were coding genes (Figure 4E, Supplementary Table 4). Gene ontology (GO) and Kyoto Encyclopedia of Genes and Genomes (KEGG) functional enrichment analyses indicated that YTHDF1-binding genes were involved in the MAPK signaling pathway, Wnt signaling pathway, mitotic nuclear division, and regulation of mRNA processing (Figure 4F). The cumulative distribution analysis indicated that there was no significant difference between the YTHDF1-targeted genes and the non YTHDF1-targeted genes at the transcription level (Figure 4G), which was consistent with previous findings supporting the role of YTHDF1 in the mRNA translational control.

As it is well known that YTHDF1 is an m6A reader protein, we applied MeRIP-seq in PC-3 cells to identify m6A modification genes. A total of 35,906 peaks were identified in 12,497 genes (Supplementary Table 4). The most common m6A motif, GGAC, was significantly enriched in the m6A peaks (Figure 4H), suggesting that immunoprecipitation of the m6A modification transcripts was successful. Up to 63.9% of m6A modifications were located on the transcripts of protein-coding genes (Figure 4I), and m6A modifications were predominantly abundant in mRNA open reading frames and 3ʹUTR regions (Figure 4J, K). Since several studies have demonstrated that YTHDF1 regulates gene translation, we performed TMT labelling proteomic analysis in YTHDF1-KO and negative control PC-3 cells. The results showed that there were 610 proteins upregulated and 1,473 proteins were downregulated (Supplementary Table 4).

**Figure 5:**
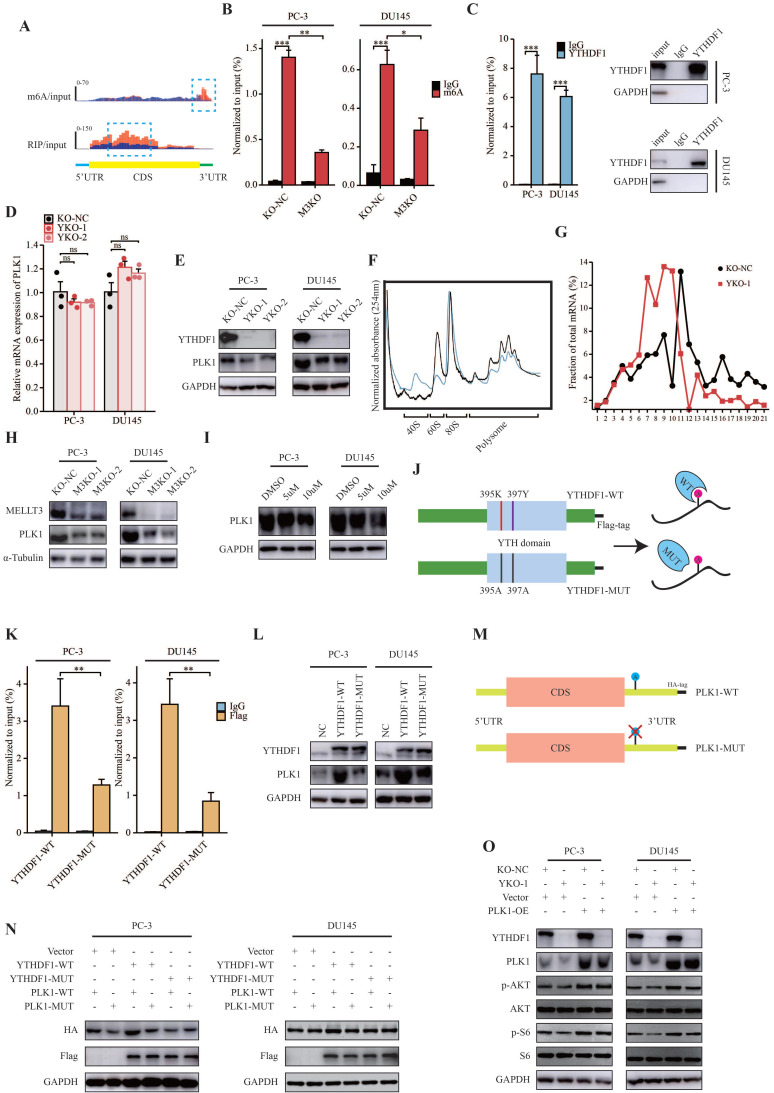
** YTHDF1 directly targeted PLK1 and regulated PLK1 translational efficiency in an m6A dependent manner. (A)** Distribution of m6A peaks and YTHDF1-binding peaks across PLK1 transcripts. **(B)** MeRIP-qPCR analysis validated m6A levels of PLK1 mRNA in PC-3 and DU145 cells. **(C)** RIP-qPCR analysis validated the interaction between YTHDF1 and PLK1 mRNA in PC-3 and DU145 cells. **(D)** qPCR analysis in PC-3 and DU145 cells knockout of YTHDF1. **(E)** Western blot analysis of YTHDF1-KO prostate cancer cells. **(F-G)** Polysome profiling of PC-3 KO-NC and YTHDF1-KO cells. **(H)** Western blot analysis of PLK1 in METTL3-KO prostate cancer cells. **(I)** Western blot analysis of PLK1 in prostate cancer cells treated with the m6A inhibitor DAA. **(J)** Schematic description showing wild-type (YTHDF1-WT) and mutant (YTHDF1-MUT) YTHDF1 constructs. **(K)** Flag-specific RIP-qPCR analysis of PC-3 and DU145 cells. **(L)** Western blot analysis of PLK1 in prostate cancer cells transfected with YTHDF1-WT or YTHDF1-MUT vector. **(M)** Schematic description showing the wild-type (PLK1-WT) and mutant (PLK1-MUT) PLK1 constructs. **(N)** Western blot analysis of HA-tagged PLK1 in PC-3 and DU145 cells transfected with the empty vector, Flag-tagged YTHDF1-WT or Flag-tagged YTHDF1-MUT, and HA-tagged PLK1-WT or HA-tagged PLK1-MUT. **(O)** Western blot analysis of YTHDF1-KO prostate cancer cells overexpressing PLK1. Data were indicated as mean ± standard deviation, ns P ≥ 0.05, * P < 0.05, ** P < 0.01, *** P < 0.001.

To identify the direct targets of YTHDF1, we then overlapped the genes identified by MeRIP-seq, RIP-seq, and TMT proteomic analysis, and the results showed that 118 genes were significantly downregulated at the protein level. Of those 118 genes, 81 genes were not affected at the mRNA expression level (Figure 4L, Supplementary Figure S4A). Through literature search, we screened several genes significantly associated with prostate cancer progression: ADRB2, LETM1, MED19, GTSE1, PLK1, PML, and KDM6B (Figure 5A, Supplementary Figure S4B‒G). In addition, RIP-qPCR assays were performed to verify the binding of these gene transcripts to YTHDF1 (Supplementary Figure S4H). Among the seven downregulated genes, we found that PLK1 was an important mitogenic gene involved in DNA replication and cell cycle (Figure 4M), which was consistent with our functional enrichment analysis results. Therefore, we selected PLK1 as YTHDF1 direct downstream target for further validation.

### PLK1 was the m6A modification target of YTHDF1

First, Integrative Genomics Viewer (IGV) analysis revealed that the m6A peaks among PLK1 were located in the 3ʹUTR region according to MeRIP-seq, and YTHDF1 bound to PLK1 mRNA (Figure 5A). The results of MeRIP-qPCR confirmed that PLK1 transcripts had m6A modifications in PC-3 and DU145 cells, which could be suppressed by METTL3-KO (Figure 5B). Additionally, YTHDF1 specific RIP-qPCR results demonstrated an interaction between PLK1 mRNA and YTHDF1 in PC-3 and DU145 cells (Figure 5C). Furthermore, we evaluated the role of YTHDF1-KO in the transcriptional and translational efficiency of PLK1. Consistent with previous studies, when YTHDF1 was knocked out in prostate cancer cells, the RNA level of PLK1 was relatively unchanged, whereas a prominent decrease in the protein level was observed (Figure 5D, E). Polysome profiling indicated that the translational efficiency of PLK1 mRNA was significantly impaired in YTHDF1-KO PC-3 cells (Figure 5F, G). In addition, the protein level of PLK1 was downregulated in METTL3-KO prostate cancer cells and in cells treated with the m6A inhibitor DAA (Figure 5H, I). These data indicated that PLK1 is an m6A modification target of YTHDF1.

### YTHDF1 regulated the translational effect of PLK1 in a m6A-dependent manner

Next, we questioned whether the YTHDF1-regulated PLK1 translation efficiency was m6A modification-dependent. Thus, a Flag-tagged mutant YTHDF1 vector (YTHDF1-MUT) was constructed with two key amino acid mutations (K395A and Y397A) to impair its binding ability with mRNA through its m6A binding pockets in the YTH domain (Figure 5J). Subsequently, Flag-specific RIP-qPCR results revealed that PLK1 mRNA was effectively immunoprecipitated in PC-3 and DU145 cells transfected with wild-type YTHDF1 (YTHDF1-WT), whereas the interaction between PLK1 and YTHDF1-MUT was significantly inhibited (Figure 5K). Western blotting indicated that YTHDF1-WT overexpression significantly induced PLK1 protein expression, whereas YTHDF1-MUT displayed only a slight effect on PLK1 protein expression (Figure 5L).

To further elucidate the mechanisms of m6A modification of PLK1 mRNA, we constructed HA-tagged PLK1 mutants (PLK1-MUT) with mutations in m6A peaks in the 3ʹUTR region (Figure 5M). Western blotting indicated that compared with wild-type PLK1 (PLK1-WT), PLK1-MUT protein expression showed no response to YTHDF1-WT overexpression (Figure 5N). Compared to YTHDF1-WT, YTHDF1-MUT had no effect on HA-tagged PLK1 protein expression (Figure 5N). In summary, these results demonstrate that the translational efficiency of PLK1 mRNA regulated by YTHDF1 is dependent on m6A modifications.

### YTHDF1 regulated PI3K/AKT signaling pathway through PLK1 in prostate cancer

Because of the role of PLK1 in the regulation of progression of prostate cancer through the activation of the PI3K/AKT signaling pathway, we hypothesized that YTHDF1 promotes PLK1 mRNA translational efficiency in activating PI3K/AKT signaling, thereby regulating prostate cancer tumorigenesis and metastasis. Western blotting revealed that YTHDF1-KO in PC-3 and DU145 cells inhibited the PI3K/AKT signaling pathway; however, this effect could be rescued by the overexpression of PLK1 in YTHDF1-KO cells (Figure 5O).

### YTHDF1 facilitated prostate cancer progression through regulating PLK1 expression

To further investigate the role of PLK1 in prostate cancer, we analyzed the expression of PLK1 in TCGA data. Results showed that PLK1 was dysregulated in many tumors (Supplementary Figure S5A). Moreover, upregulated PLK1 was associated with a higher pathologic T stage, N stage, and Gleason scores, and high expression of PLK1 was indicative of a poor prognosis in prostate cancer (Supplementary Figure S5B‒F). These results suggested that PLK1 was a prognostic indicator in prostate cancer.

**Figure 6:**
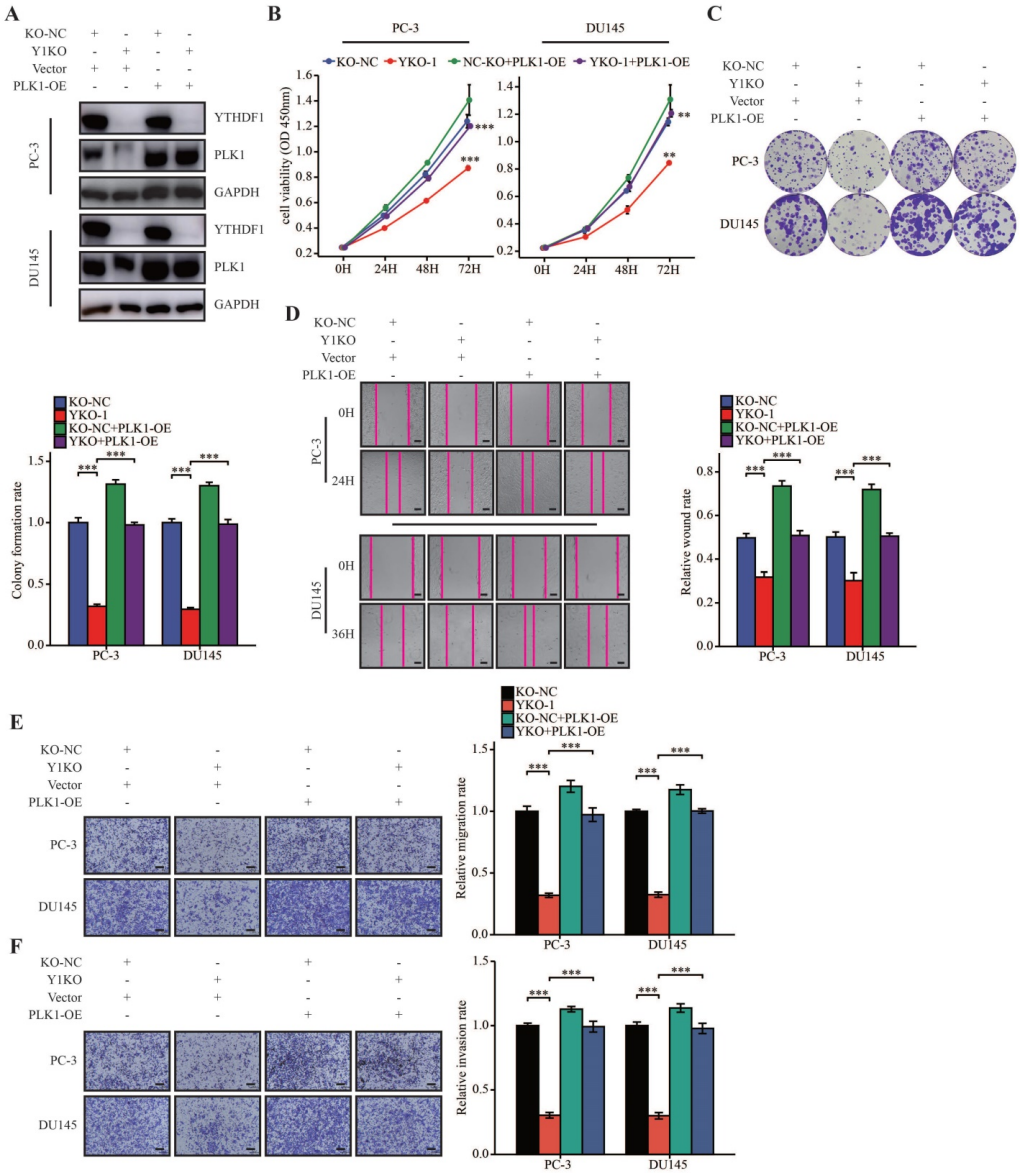
** YTHDF1 promoted prostate cancer progression through regulating PLK1 expression. (A)** Western blot analysis of YTHDF1 and PLK1 expression in YTHDF1-knockout prostate cancer cells transfected with either the PLK1 overexpression vector or empty vector control. **(B)** Viability of transfected PC-3 and DU145 cells, as described in the methods section. **(C)** Colony formation assay of PC-3 and DU145 cells, as described transfected. **(D)** Wound healing assays were conducted in transfected PC-3 and DU145 cells, as described in the methods section (Scale bar: 50 μm). **(E‒F)** Transwell migration and invasion assays were performed in PC-3 and DU145 cells as described in the methods section (Scale bar: 50 μm). Data were indicated as mean ± standard deviation, ns P ≥ 0.05, * P < 0.05, ** P < 0.01, *** P < 0.001.

To further elucidate the oncogenic role of YTHDF1/PLK1 axis prostate cancer cells, we overexpressed PLK1 in YTHDF1-deficient PC-3 and DU145 cells (Figure 6A). YTHDF1-KO impaired cell proliferation and colony formation, whereas PLK1 overexpression reversed this phenotype (Figure 6B, C). Indeed, overexpression of PLK1 increased cell migration and invasion in YTHDF1-deficiency prostate cancer cells (Figure 6D‒F). To sum up, we demonstrated that YTHDF1 promoted prostate cancer progression by increasing the translational efficiency of PLK1 in an m6A dependent manner.

### ELK1 directly bind to promoter region of YTHDF1 to regulate its transcriptional activation

**Figure 7:**
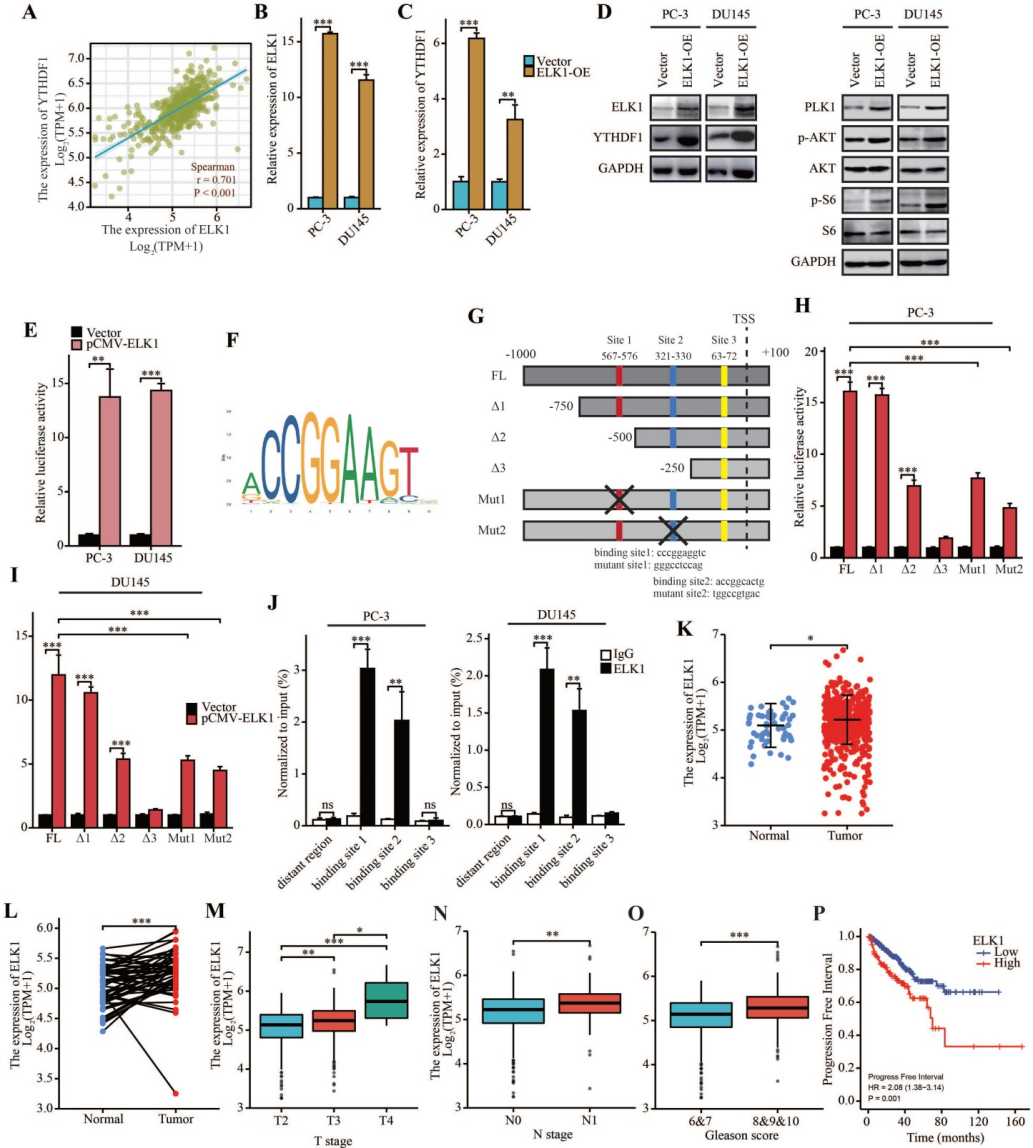
** ELK1 induced upregulation of YTHDF1 and promoted prostate cancer progression. (A)** Correlation between YTHDF1 and ELK1 expression according to TCGA database. **(B)** qPCR analysis of ELK1 expression in ELK1 overexpression PC-3 and DU145 cells. **(C)** qPCR analysis of YTHDF1 expression in ELK1 overexpression PC-3 and DU145 cells. **(D)** Western blot analysis of ELK1, YTHDF1, PLK1, p-AKT, AKT, p-S6, and S6 expression in ELK1 overexpression PC-3 and DU145 cells. **(E)** Relative luciferase reporter assays in PC-3 and DU145 cells after the co-transfection of plasmid constructs containing the YTHDF1 promoter with a ELK1 overexpressing construct. **(F)** The DNA motif for ELK1 was obtained from JASPAR. **(G)** ELK1 binding sites in YTHDF1 promoter were predicted by JASPAR. **(H-I)** Relative luciferase reporter assays in prostate cancer cells after the co-transfection of a series of truncated and mutated YTHDF1 promoter with a ELK1 overexpressing construct. **(J)** ChIP assay revealed the direct interactions between ELK1 and YTHDF1 promoter in prostate cancer cells. **(K)** RNA levels of ELK1 in prostate cancer and adjacent normal prostate tissues in TCGA database. **(L)** RNA levels of ELK1 in prostate cancer and paired adjacent normal prostate tissues in TCGA database. **(M)** Correlation between T stage and ELK1 expression. **(N)** Correlation between N stage and ELK1 expression. **(O)** Correlation between the Gleason score and ELK1 expression. **(P)** Kaplan-Meier analysis of prostate cancer patients for correlation between ELK1 expression and progression free interval. Data were indicated as mean ± standard deviation, ns P ≥ 0.05, * P < 0.05, ** P < 0.01, *** P < 0.001.

Next, we investigated the cause of YTHDF1 dysregulation in prostate cancer. Transcription factors are involved in regulation of transcriptional activation of genes. Thus, we searched for transcription factors most significantly correlated with YTHDF1 expression by analyzing TCGA database. Importantly, ETS transcription factor ELK1, which is known to be dysregulated in multiple types of tumors, was significantly related to YTHDF1 expression (Figure 7A). Therefore, we suspected that ELK1 may be involved in the regulation of YTHDF1 expression. qPCR analysis and Western blot analysis showed that YTHDF1 expression was upregulated in ELK1-overexpressing prostate cancer cells (Figure 7B-D), and dual-luciferase reporter assays suggested that ELK1 transduction enhanced the transcriptional activity of the luciferase reporter flanked by the YTHDF1 promoter in both PC-3 and DU145 cells, indicating that ELK1 may transactivate YTHDF1 expression (Figure 7E).

To further clarify the related regulatory mechanisms, the promoter sequence of YTHDF1 was analyzed using JASPAR (http://jaspar.genereg.net/), and three putative ELK1 binding motifs were found (Figure 7F, G). Then, a series of luciferase reporter plasmids harboring truncated or mutated YTHDF1 promoter sequences was constructed and transfected into PC-3 and DU145 cells. The results indicated that -750 bp to -250 bp was essential for ELK1-induced expression of the luciferase reporter. Furthermore, site-directed mutagenesis of the YTHDF1 promoter showed that putative binding site 1 and 2 in the promoter were indispensable for ELK1 binding (Figure 7H-I). In addition, ChIP-qPCR assays suggested that ELK1 directly bound to the binding site 1 and 2 of YTHDF1 promoter region in prostate cancer cells (Figure 7J). Thus, our results demonstrated that YTHDF1 transcription was activated by aberrant ELK1 in prostate cancer.

### ELK1 is a prognostic indicator and facilitates prostate cancer progression

To further elucidate the role of ELK1 in prostate cancer, we investigate ELK1 expression by analyzing TCGA database. Result showed that ELK1 was upregulated in prostate cancer in cancer tissue compared with normal prostate tissue, and a significant increase was also observed in prostate cancer compared to paired normal prostate tissue (Figure 7K, L). In addition, ELK1 expression was enhanced in advanced prostate cancer (Figure 7M-O). Kaplan-Meier survival analysis indicated that high ELK1 expression was correlated with poor PFI (Figure 7P). These results demonstrated that ELK1 is a prognostic indicator in prostate cancer.

To further explore the pathological roles of ELK1 in prostate cancer, we constructed ELK1 overexpression prostate cancer cells by transfection with pCMV-ELK1. ELK1-overexpression promoted cell proliferation and colony-forming ability of prostate cancer cells (Supplementary Figure 6A, B). Moreover, upregulated ELK1 induced migration and invasion of prostate cancer cells (Supplementary Figure 6C-E). To sum up, these results demonstrated that the oncogenic gene ELK1 enhanced the transcriptional activation of YTHDF1 by binding to its promoter region in prostate cancer.

## Discussion

Dysregulation of m6A methylation is closely associated with tumorigenesis in multiple cancers. In this study we showed the upregulation of YTHDF1 in prostate cancer and its role in regulating prostate cancer tumorigenesis and metastasis through the induction of PLK1 mRNA translational efficiency in an m6A modification-dependent manner.

Blocking m6A enzyme activity may represent a strategy to inhibit cancer development. For instance, FB23-2, an m6A-RNA demethylase FTO inhibitor, impairs acute myeloid leukemia cell proliferation and induces apoptosis 31. R-2HG, another FTO inhibitor, suppresses the stability of MYC/CEBPA transcripts by increasing global m6A modification, thereby decreasing relative signaling pathway activation 32. Therefore, inhibitors of m6A enzymes have shown great potential for tumor treatment 33.

Several studies have elucidated the biological functions of m6A methylation in prostate cancer. For example, YTHDF2 was upregulated in prostate cancer, and YTHDF2 knockdown impaired prostate cancer proliferation, migration, and invasion. Mechanistically, YTHDF2 inhibited AKT phosphorylation by decreasing the stability of LHPP and NKX3-1 mRNA in an m6A dependent manner 34. In addition, METTL3-mediated m6A modified PCAT6 was upregulated in an IGF2BP2 dependent manner. High expression of PCAT6 enhanced IGF1R mRNA stability, thus contributing to prostate cancer tumorigenesis and metastasis 35. Nevertheless, the role of m6A methylation in prostate cancer remains largely unknown. Here, we identified YTHDF1 as a poor prognostic indicator for prostate cancer progression. In addition, overexpression of YTHDF1 facilitated prostate cancer proliferation, migration, and invasion *in vitro* and *in vivo*. We obtained a comprehensive view of YTHDF1-mediated translation and gene regulation in prostate cancer by integrating RNA-seq, RIP-seq, m6A-seq, and TMT proteomic analyses. We showed that YTHDF1 mediated prostate carcinogenesis by increasing the protein level of PLK1 and activating the PI3K/AKT signaling pathway. Moreover, YTHDF1 recognized and interacted with the m6A modification of the 3ʹUTR of PLK1 mRNA, thereby facilitating the translational efficiency of PLK1.

The Polo-like kinase family consists of five proteins, PLK1-5, of which the molecular function of PLK1 is the most investigated 36. PLK1 plays a prominent role in cell cycle. PLK1 regulates centrosome localization of Aurora A and contributes to centrosome maturation during the G2 phase 37. Moreover, PLK1 inhibits Tyr-15 phosphorylation of CDK1 by activating CDC25C phosphatase, thereby contributes to the activation of Cyclin B/CDK1 complexes 38, 39. An increasing number of studies have shown that PLK1 is dysregulated in tumor tissues 40-43. Furthermore, targeting PLK1 activation may be a therapeutic approach for cancer treatment 44-46. However, the molecular mechanism by which PLK1 regulates prostate cancer progression has not yet been elucidated. Research has demonstrated that PLK1 could affect androgen receptor elevation, lipid metabolism, and response to androgen signaling inhibitors in prostate cancer by regulating the PI3K/AKT signaling pathway 47. Therefore, we hypothesized that YTHDF1 could affect PI3K/AKT pathway activation by regulating the protein level of PLK1, thereby promoting prostate cancer tumorigenesis and metastasis. Research has shown that METTL3-mediated PLK1 m6A modification may regulate cell cycle progression of dental pulp stem cells 48. However, m6A modification of the 3ʹUTR region of PLK1 has not been clearly elucidated. In our studies, we suggested that m6A modification of PLK1 mRNA could be recognized by m6A “reader” YTHDF1, which then induced PLK1 translational rate in prostate cancer, thus promoting prostate cancer progression.

Finally, we demonstrated that ELK1 contributed to YTHDF1 overexpression in prostate cancer. Although some articles reported that ELK1 regulated AR transcription and thus affected the progression of AR-positive prostate cancer cells 29, the role of ELK1 in AR-negative prostate cancer have not been fully elucidated. Our data demonstrated that overexpression of ELK1 in prostate cancer cells contributed to the transcriptional activation of YTHDF1, further resulting in aberrant regulation of PLK1/PI3K/AKT axis.

## Conclusion

**Figure 8:**
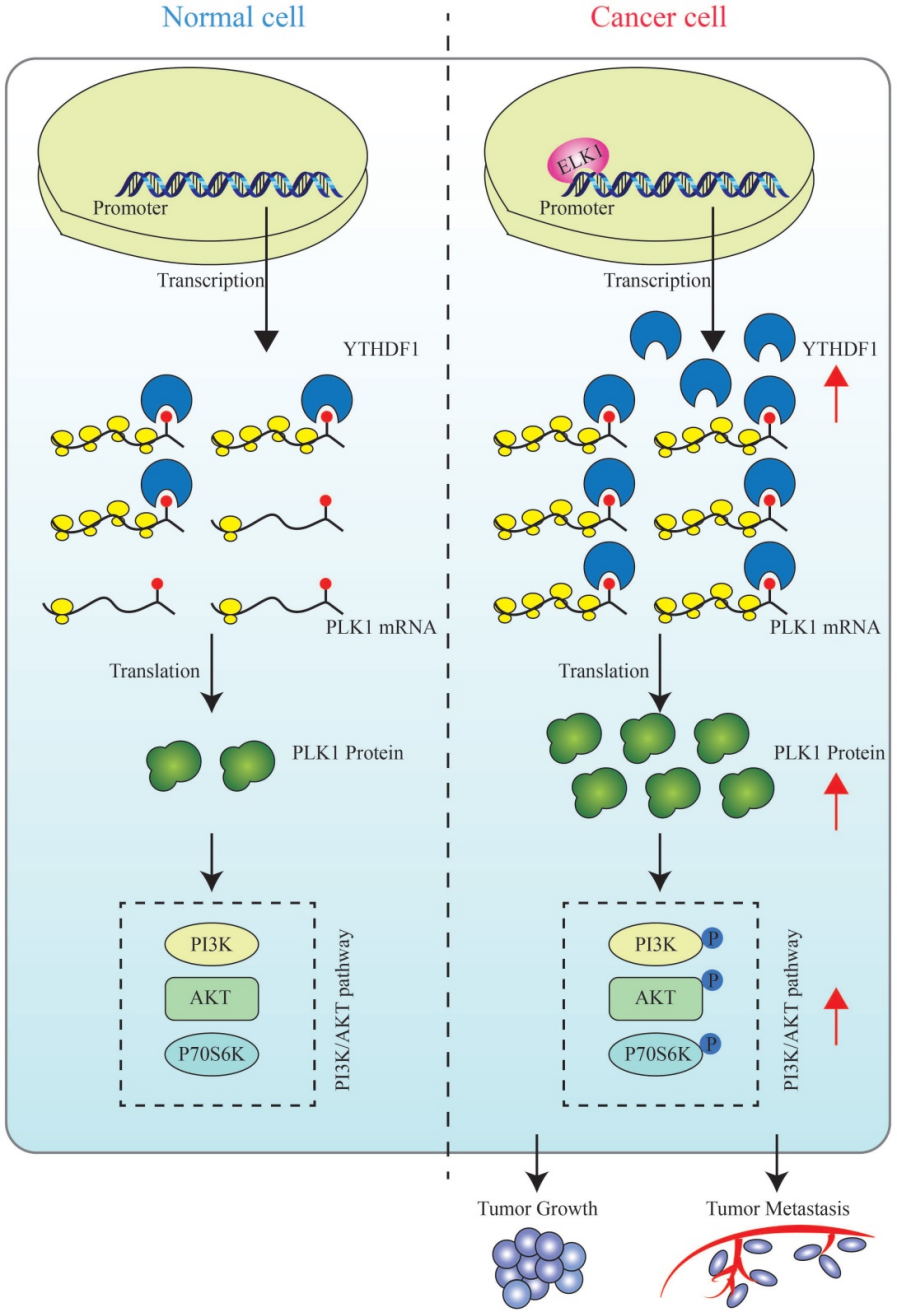
** A schematic diagram for the role of YTHDF1 in prostate cancer progression.** ELK1 directly interacts with the promoter region of YTHDF1 to regulate the transcriptional activation of YTHDF1. Dysregulated YTHDF1 promotes PLK1 translational efficiency by recognizing and directly binding to m6A modification of the 3ʹUTR region of PLK1 mRNA, thereby regulating the activation of the PI3K/AKT signaling pathway and contributing to prostate cancer tumorigenesis and metastasis.

Our study identified PLK1, a key factor in the cell cycle, as the direct target of YTHDF1 in prostate cancer cells. This finding represented a significant contribution in elucidating the mechanism of m6A modification in prostate cancer development. Our data suggested that ELK1-activated YTHDF1 controlled the translational efficiency of PLK1 in an m6A-dependent manner and affected the activation of the PI3K/AKT signaling pathway (Figure 8). Therefore, targeting YTHDF1 may be a promising therapeutic strategy for prostate cancer therapy.

## Supplementary Material

Supplementary figures and tables 1-3, 5.

Supplementary table 4.

## Figures and Tables

**Table 1:** Patients' information in the Tumor microarray

Characteristic	Low YTHDF1 exp	High YTHDF1 exp	p
n	46	54	
T stage, n (%)			< 0.001
T2	15 (16%)	4 (4.3%)	
T3	28 (29.8%)	30 (31.9%)	
T4	2 (2.1%)	15 (16%)	
N stage, n (%)			0.148
N0	36 (36%)	34 (34%)	
N1	10 (10%)	20 (20%)	
Gleason scores, n (%)			0.173
6	3 (3%)	3 (3%)	
7	28 (28%)	21 (21%)	
8	8 (8%)	14 (14%)	
9	7 (7%)	14 (14%)	
10	0 (0%)	2 (2%)	
Age, mean ± SD	69.93 ± 5.68	71.83 ± 6.36	0.121

**Table 2:** Univariate and multivariate analysis of factors associated with OS using Cox regression.

Characteristics	Univariate analysis		Multivariate analysis	
	Hazard ratio (95% CI)	P value	Hazard ratio (95% CI)	P value
T stage (T3&T4 vs T2)	3.239 (1.314-7.986)	0.011	2.166 (0.822-5.705)	0.118
N stage (N1 vs N0)	2.151 (0.702-6.586)	0.180		
Gleason score (8&9&10 vs 6&7)	2.311 (1.399-3.817)	0.001	2.053 (1.193-3.532)	0.009
YTHDF1 (High vs Low)	3.295 (1.338-8.117)	0.010	3.386 (1.362-8.418)	0.009

## Abbreviations

m6A: N6-methyladenosine
YTHDF1: YTH m6A RNA-binding protein 1
PLK1: Polo-like kinase 1
ADT: Androgen deprivation therapy
OS: Overall survival
CRPC: Castration-resistant prostate cancer
WTAP: Wilms tumor 1 associated protein
FZD7: Frizzled class receptor 7
EIF3C: Eukaryotic translation initiation factor 3 subunit C
MEM: Minimum Essential Media
FBS: Fetal bovine serum
SDS-PAGE: Sodium dodecyl sulfate-polyacrylamide gel electrophoresis
PVDF: Polyvinylidene fluoride
CHX: Cycloheximide
PFI: Progression Free Interval
IHC: Immunohistochemistry
TCGA: The Cancer Genome Atlas
GSEA: Gene set enrichment analysis
GO: Gene ontology
KEGG: Kyoto Encyclopedia of Genes and Genomes
IGV: Integrative Genomics Viewer
