# Supplementary material for: ELK1-mediated YTHDF1 drives prostate cancer progression by facilitating the translation of Polo-like kinase 1 in an m6A dependent manner
Source: Int J Biol Sci. 2022 Oct 18;18(16):6145–62. doi: 10.7150/ijbs.75063 (PMC9682537; doi:10.7150/ijbs.75063)
Supplement: Supplementary file 1 — Supplementary figures and tables 1-3, 5. [file ijbsv18p6145s1.pdf]

Supplementary Materials

Legends

Supplementary Figure 1. Immunohistochemical (IHC) images of YTHDF1 expression in tumor microarrays.

Supplementary Figure 2. YTHDF1 expression in prostate cancer cell lines and normal prostate epithelial cell line.

(A) qPCR analysis of YTHDF1 in PC-3, DU145, and RWPE-1 cells. (**B)** Western blot analysis of YTHDF1 in PC-3, DU145, and RWPE-1 cells. Data were indicated as mean ± SD, ns $P \geq 0.05$, * $P < 0.05$, ** $P < 0.01$, *** $P < 0.001$.

Supplementary Figure 3. YTHDF1 knockdown inhibited prostate cancer cell proliferation, migration, and invasion.

(A) RT-qPCR analysis of YTHDF1-knockdown PC-3 and DU145 cells. (**B)** Western blot analysis of YTHDF1-knockdown PC-3 and DU145 cells. (**C-D)** Analysis of cell viability in YTHDF1-knockdown PC-3 and DU145 cells using CCK-8 assays. (**E)** Colony formation assay was conducted to determine YTHDF1-knockdown prostate cancer cell colony formation ability. (**F)** Wound healing assay showing migration ability of YTHDF1-knockdown prostate cancer cells (Scale bar: 50 μm). (**G-H)** Transwell migration and transwell invasion assay were conducted to determine the migration and invasion capacity of prostate cancer cells stably knockdown of YTHDF1 (Scale bar: 50 μm). Data were indicated as mean ± SD, ns $P \geq 0.05$, * $P < 0.05$, ** $P < 0.01$, *** $P < 0.001$.

Supplementary Figure 4. Distribution of m6A modification peaks and YTHDF1-binding peaks

across transcripts.

(A) Overlapping analysis of genes identified by m6A-seq, RIP-seq, RNA-seq and proteomic analysis. **(B)** M6A peaks and YTHDF1-binding peaks at ADRB2 mRNAs. (**C**) M6A peaks and YTHDF1-binding peaks at LETM1 mRNAs. (**D**) M6A peaks and YTHDF1-binding peaks at MED19 mRNAs. (**E**) M6A peaks and YTHDF1-binding peaks at GTSE1 mRNAs. (**F**) M6A peaks and YTHDF1-binding peaks at PML mRNAs. (**G**) M6A peaks and YTHDF1-binding peaks at KDM6B mRNAs. (**H**) RIP-qPCR analysis of GAPDH, ADRB2, LETM1, MED19, GTSE1, PML, KDM6B, and PLK1. Data were indicated as mean ± SD, ns P ≥ 0.05, * P < 0.05, ** P < 0.01, *** P < 0.001.

Supplementary Figure 5. PLK1 was up-regulated in prostate cancer and indicated a poor prognosis.

(A) Expression levels of PLK1 in TCGA cancers and adjacent normal tissues. (**B**) The correlation between T stage and PLK1 expression in TCGA database. (**C**) The correlation between N stage and PLK1 expression in TCGA database. (**D**) The correlation between Gleason scores and PLK1 expression in TCGA database. (**E**) Kaplan-Meier analysis of prostate cancer patients for the correlations between PLK1 expression and Overall Survival (OS). (**F**) Kaplan-Meier analysis of prostate cancer patients for the correlations between PLK1 expression and Progression Free Interval (PFI). Data were indicated as mean ± SD, ns P ≥ 0.05, * P < 0.05, ** P < 0.01, *** P < 0.001.

Supplementary Figure 6. Overexpression of ELK1 regulated prostate cancer progression.

(**A**) Cell viability analysis of ELK1-overexpression prostate cancer cells using CCK-8 assays. (**B**) Analysis of colony formation ability in ELK1-overexpression prostate cancer cells using colony

formation assays. **(C)** Wound-healing assay was performed to determine the migration of ELK1-overexpression PC-3 and DU145 cells (Scale bar: 50 μm). **(D-E)** Transwell migration and invasion assays were conducted to determine the migration and invasion capacity of stable ELK1 overexpressing prostate cancer cells (Scale bar: 50 μm). Data were indicated as mean ± standard deviation, ns $P \geq 0.05$, * $P < 0.05$, ** $P < 0.01$, *** $P < 0.001$.

Figure S1

A

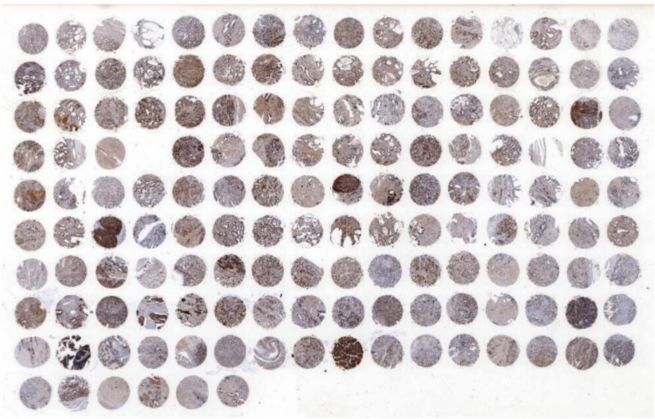

B

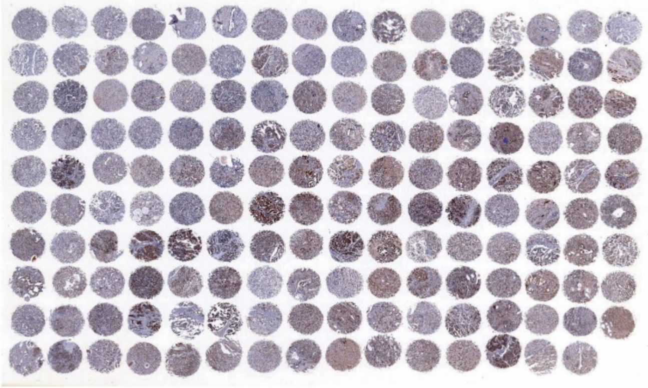

Figure S2

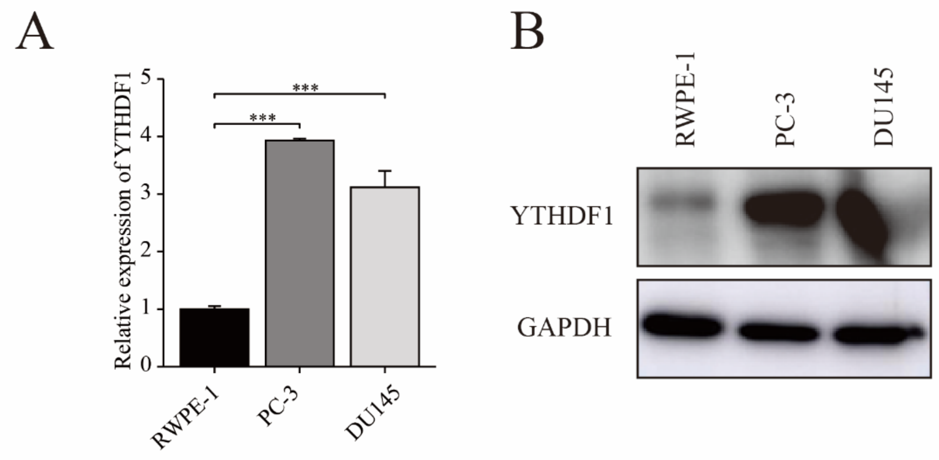
A
B
Relative expression of YTHDF1
5
4
3
2
1
0
***
***
RWPE-1
PC-3
DU145
RWPE-1
PC-3
DU145
YTHDF1
GAPDH

Figure S3

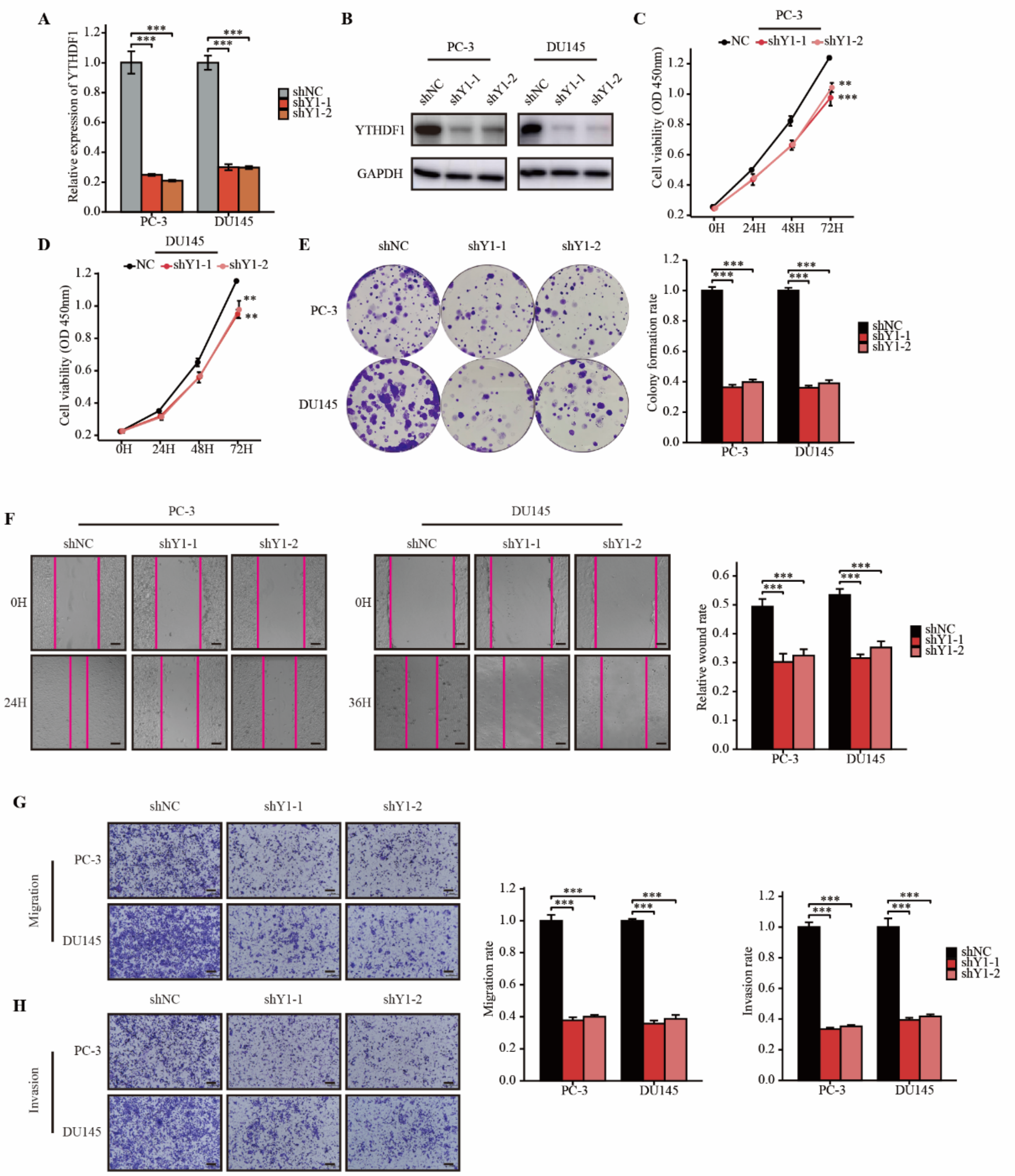

A
Relative expression of YTHDF1
shNC
shY1-1
shY1-2
PC-3
DU145
B
PC-3
DU145
shNC
shY1-1
shY1-2
YTHDF1
GAPDH
C
PC-3
NC
shY1-1
shY1-2
Cell viability (OD 450nm)
0H
24H
48H
72H
D
DU145
E
shNC
shY1-1
shY1-2
PC-3
DU145
Colony formation rate
F
PC-3
DU145
0H
24H
36H
Relative wound rate
G
Migration
Migration rate
H
Invasion
Invasion rate

Figure S4

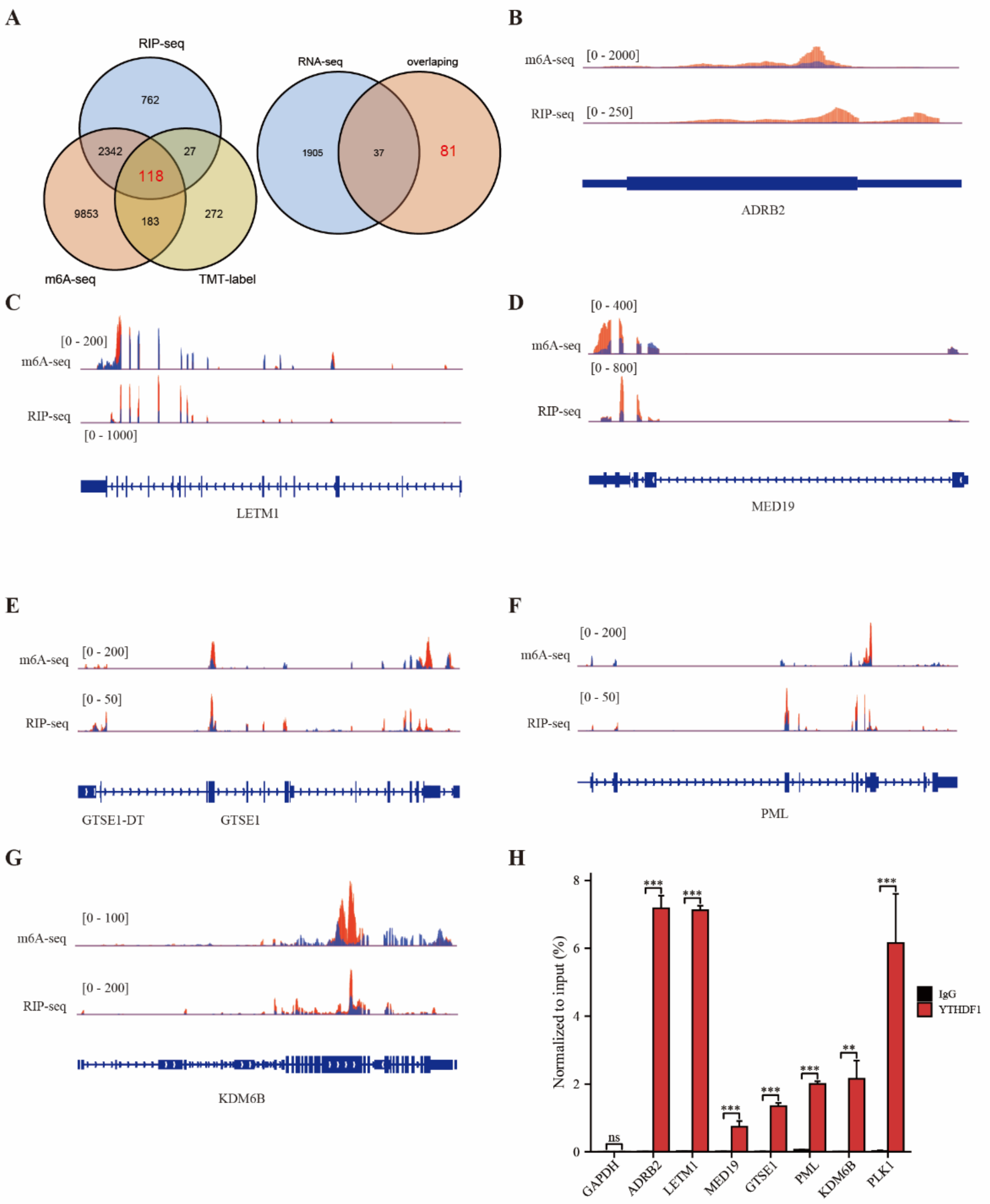
A
RIP-seq
762
2342
27
118
9853
183
272
m6A-seq
TMT-label
RNA-seq
overlaping
1905
37
81
B
m6A-seq [0 - 2000]
RIP-seq [0 - 250]
ADRB2
C
[0 - 200]
m6A-seq
RIP-seq
[0 - 1000]
LETM1
D
[0 - 400]
m6A-seq
[0 - 800]
RIP-seq
MED19
E
m6A-seq [0 - 200]
[0 - 50]
RIP-seq
GTSE1-DT
GTSE1
F
[0 - 200]
m6A-seq
[0 - 50]
RIP-seq
PML
G
[0 - 100]
m6A-seq
[0 - 200]
RIP-seq
KDM6B
H
Normalized to input (%)
IgG
YTHDF1
ns
***
**
GAPDH
ADRB2
LETM1
MED19
GTSE1
PML
KDM6B
PLK1

Figure S5

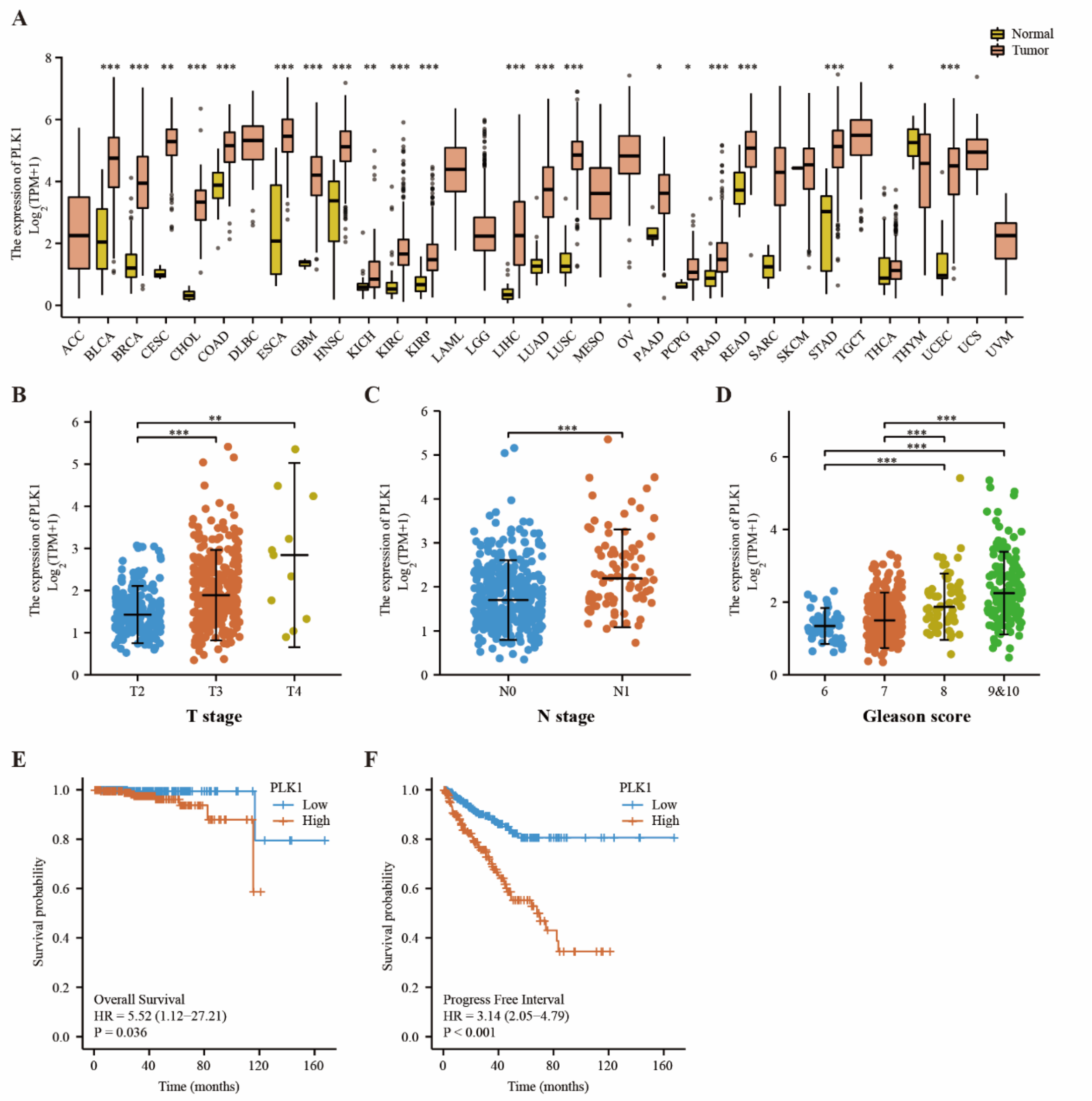

A
Normal
Tumor
The expression of PLK1 Log2(TPM+1)
ACC BLCA BRCA CESC CHOL COAD DLBC ESCA GBM HNSC KICH KIRC KIRP LAML LGG LIHC LUAD LUSC MESO OV PAAD PCPG PRAD READ SARC SKCM STAD TGCT THCA THYM UCEC UCS UVM
B
T2 T3 T4
T stage
C
N0 N1
N stage
D
6 7 8 9&10
Gleason score
E
PLK1
Low
High
Survival probability
Overall Survival
HR = 5.52 (1.12–27.21)
P = 0.036
Time (months)
F
PLK1
Low
High
Survival probability
Progress Free Interval
HR = 3.14 (2.05–4.79)
P < 0.001
Time (months)

Figure S6

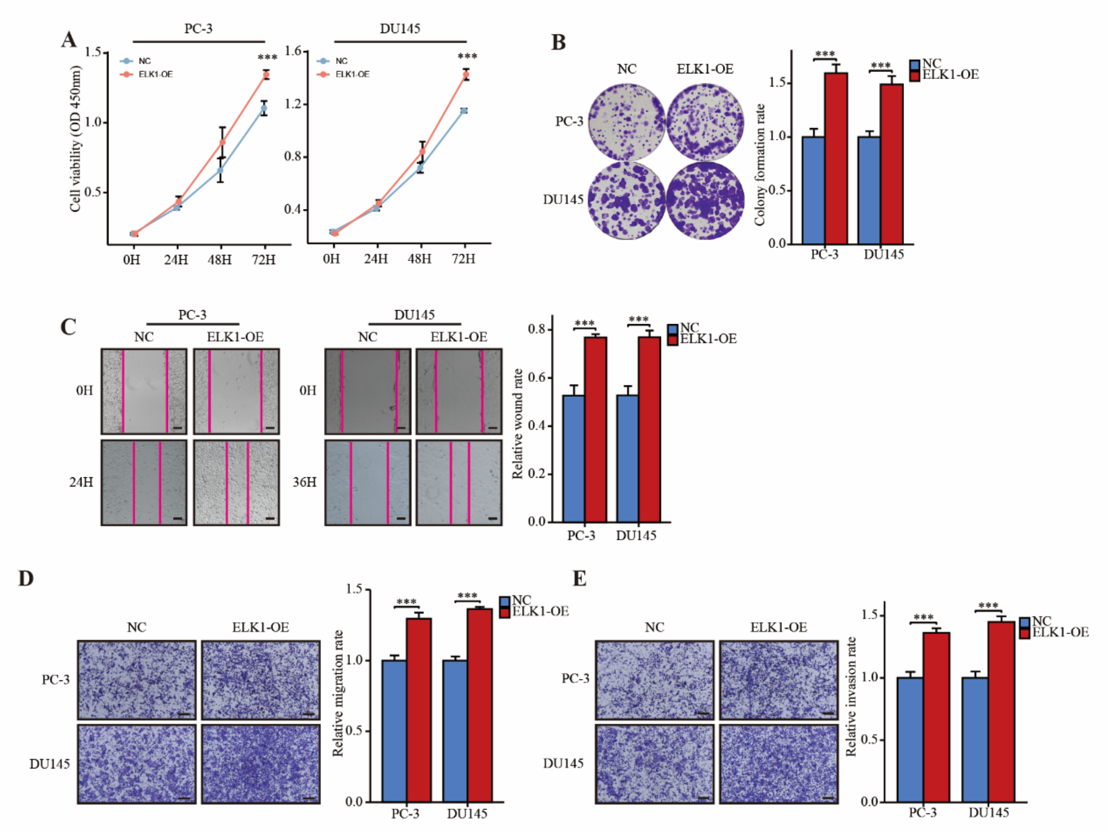

A
PC-3
DU145
NC
ELK1-OE
Cell viability (OD 450nm)
0H
24H
48H
72H
***
B
NC
ELK1-OE
PC-3
DU145
Colony formation rate
***
C
PC-3
DU145
NC
ELK1-OE
0H
24H
36H
Relative wound rate
***
D
NC
ELK1-OE
PC-3
DU145
Relative migration rate
***
E
NC
ELK1-OE
PC-3
DU145
Relative invasion rate
***

Supplementary Table 1. Primers for RT-qPCR

Name	Primer Sequence
YTHDF1-qF	GGGGACAAGTGGGTCTCAAG
YTHDF1-qR	AGGGTGTCGCTGTGAAAGC
GAPDH-qF	CTGGGCTACACTGAGCACC
GAPDH-qR	AAGTGGTCGTTGAGGGCAATG
PLK1-qF	CCTGCACCGAAACCGAGTTAT
PLK1-qR	CCGTCATATTCGACTTTGGTTGC
PLK1-F (m6A)	TCAAGGCCTCCTAATAGCTGCC
PLK1-R (m6A)	CCACACCCGAACATGTACAAAAA
ADRB2-qF	TGGTGTGGATTGTGTCAGGC
ADRB2-qR	GGCTTGGTTCGTGAAGAAGTC
LETM1-qF	CCGAGTGCCTTCGCATAGTG
LETM1-qR	ACTTCTCTACTACCGAGTCATCG
MED19-qF	ATGGAGAATTTCACGGCACTG
MED19-qR	ATGGAGAATTTCACGGCACTG
GTSE1-qF	CAGGGGACGTGAACATGGATG
GTSE1-qR	CAGGGGACGTGAACATGGATG
PML-qF	GGATGAAGTGCTACGCCTCG
PML-qR	GGATGAAGTGCTACGCCTCG
KDM6B-qF	CGCTGCCTCACCCATATCC
KDM6B-qR	CGCTGCCTCACCCATATCC
ELK1-qF	TCCCTGCTTCCTACGCATACA
ELK1-qR	GCTGCCACTGGATGGAAACT
Distant region-F	GGCAGATGACAGTTCTCTGCAG
Distant region-R	CGGCATGACATCCCCCA
Binding site 1-F	CCTCCTGGTGATGAAATCGG
Binding site 1-R	CTTCAGCCGCAGCGGA
Binding site 2-F	CTGTCGGACCAGAACAGCGTTT
Binding site 2-R	CACCCCCGACGCGCTA
Binding site 3-F	TGAGAGCGAAATCCATCCCG
Binding site 3-R	GAGGTGACACCAAGTAGCAGC

Supplementary Table 2. Antibodies used in the study

Name	Company	Catalog Number
YTHDF1	Abcam	Ab220162
YTHDF1	Proteintech	17479-1-AP
GAPDH	Cell Signaling Technology	5174
METTL3	Abcam	Ab195352
β -Tubulin	Cell Signaling Technology	5666
PLK1	Cell Signaling Technology	4513
Flag	Cell Signaling Technology	2368
HA	Cell Signaling Technology	5017
p-AKT (S473)	Cell Signaling Technology	9271
AKT	Cell Signaling Technology	9272
p-S6	Cell Signaling Technology	2211
S6	Cell Signaling Technology	2217
ELK1	Abcam	Ab32106

Supplementary Table 3. Patients' information in the TCGA-PRAD database

Characteristic	Low YTHDF1 exp	High YTHDF1 exp	p
n	249	250	
T stage, n (%)			0.003
T2	110 (22.4%)	79 (16.1%)	
T3	134 (27.2%)	158 (32.1%)	
T4	2 (0.4%)	9 (1.8%)	
N stage, n (%)			0.021
N0	171 (40.1%)	176 (41.3%)	
N1	27 (6.3%)	52 (12.2%)	
M stage, n (%)			0.616
M0	222 (48.5%)	233 (50.9%)	
M1	2 (0.4%)	1 (0.2%)	
Gleason score, n (%)			0.029
6	33 (6.6%)	13 (2.6%)	
7	121 (24.2%)	126 (25.3%)	
8	30 (6%)	34 (6.8%)	
9	64 (12.8%)	74 (14.8%)	
10	1 (0.2%)	3 (0.6%)	
PSA(ng/ml), n (%)			0.581
<4	200 (45.2%)	215 (48.6%)	
>=4	11 (2.5%)	16 (3.6%)	
Primary therapy outcome, n (%)			0.239
PD	11 (2.5%)	17 (3.9%)	
SD	19 (4.3%)	10 (2.3%)	
PR	19 (4.3%)	21 (4.8%)	
CR	168 (38.4%)	173 (39.5%)	
Age, n (%)			0.136
<=60	103 (20.6%)	121 (24.2%)	
>60	146 (29.3%)	129 (25.9%)	
Age, meidan (IQR)	62 (56, 66)	61 (56, 66)	0.456

Supplementary Table 4. Sequencing result of multi-omics analysis

Supplementary Table 5. sequences of shRNA and gRNA

	**Name**	**Sequence**
**shRNA**	shYTHDF1-1	CCGGGTTCGTTACATCAGAAGGATACTCGAGTA TCCTTCTGATGTAACGAACTTTTTG
	shYTHDF1-2	CCGGCCCGAAAGAGTTTGAGTGGAACTCGAGT TCCACTCAAACTCTTTCGGGTTTTTG
**gRNA**	YTHDF1-KO-1	ATTCCATACCTCACCACCTA PAM: CGG
	YTHDF1-KO-2	AAGGAAATCCAATGGACGGC PAM: GGG
	METTL3-KO-1	GGGCTGTCACTACGGAAGGT PAM: TGG
	METTL3-KO-2	AGCATCAGTGGGCAATGTTA PAM: AGG
